# Targeting TACC3 represents a novel vulnerability in highly aggressive breast cancers with centrosome amplification

**DOI:** 10.1038/s41418-023-01140-1

**Published:** 2023-03-02

**Authors:** Ozge Saatci, Ozge Akbulut, Metin Cetin, Vitali Sikirzhytski, Meral Uner, Deniz Lengerli, Elizabeth C. O’Quinn, Martin J. Romeo, Burcu Caliskan, Erden Banoglu, Sercan Aksoy, Aysegul Uner, Ozgur Sahin

**Affiliations:** 1 Department of Drug Discovery and Biomedical Sciences, University of South Carolina, Columbia, SC 29208 USA; 2 Department of Biochemistry and Molecular Biology, Hollings Cancer Center, Medical University of South Carolina, Charleston, SC 29425 USA; 3 Department of Pathology, Faculty of Medicine, Hacettepe University, 06100 Ankara, Turkey; 4 Department of Pharmaceutical Chemistry, Faculty of Pharmacy, Gazi University, 06100 Ankara, Turkey; 5 Translational Science Laboratory, Hollings Cancer Center, Medical University of South Carolina, Charleston, SC 29425 USA; 6 Department of Medical Oncology, Hacettepe University Cancer Institute, 06100 Ankara, Turkey

**Keywords:** Oncogenes, Tumour-suppressor proteins

## Abstract

Centrosome amplification (CA) is a hallmark of cancer that is strongly associated with highly aggressive disease and worse clinical outcome. Clustering extra centrosomes is a major coping mechanism required for faithful mitosis of cancer cells with CA that would otherwise undergo mitotic catastrophe and cell death. However, its underlying molecular mechanisms have not been fully described. Furthermore, little is known about the processes and players triggering aggressiveness of cells with CA beyond mitosis. Here, we identified Transforming Acidic Coiled-Coil Containing Protein 3 (TACC3) to be overexpressed in tumors with CA, and its high expression is associated with dramatically worse clinical outcome. We demonstrated, for the first time, that TACC3 forms distinct functional interactomes regulating different processes in mitosis and interphase to ensure proliferation and survival of cancer cells with CA. Mitotic TACC3 interacts with the Kinesin Family Member C1 (KIFC1) to cluster extra centrosomes for mitotic progression, and inhibition of this interaction leads to mitotic cell death via multipolar spindle formation. Interphase TACC3 interacts with the nucleosome remodeling and deacetylase (NuRD) complex (HDAC2 and MBD2) in nucleus to inhibit the expression of key tumor suppressors (e.g., p21, p16 and APAF1) driving G1/S progression, and its inhibition blocks these interactions and causes p53-independent G1 arrest and apoptosis. Notably, inducing CA by p53 loss/mutation increases the expression of TACC3 and KIFC1 via FOXM1 and renders cancer cells highly sensitive to TACC3 inhibition. Targeting TACC3 by guide RNAs or small molecule inhibitors strongly inhibits growth of organoids and breast cancer cell line- and patient-derived xenografts with CA by induction of multipolar spindles, mitotic and G1 arrest. Altogether, our results show that TACC3 is a multifunctional driver of highly aggressive breast tumors with CA and that targeting TACC3 is a promising approach to tackle this disease.

## Introduction

Centrosome amplification (CA) is highly prevalent in cancer [1, 2], and is strongly associated with tumor progression and worse prognosis in a variety of different cancers, e.g., breast, prostate, ovarian and lung [2]. CA is associated with several oncogenic phenotypes, such as aneuploidy, increased invasiveness and stem cell overproliferation [3]. Preclinical studies demonstrated that supernumerary centrosomes can directly trigger tumor initiation in part via compensating for mutations that decrease the functionality of centrosomes [4] or by increased rates of chromatin instability via transiently formed multipolar spindles that lead to segregation errors due to defective kinetochore-spindle attachments during mitosis [5]. There are various causes of CA, including centrosome overduplication by PLK4 overexpression, cytokinesis failure or genomic aberrations, such as p53 mutations [6, 7] that are commonly observed in aggressive tumors, e.g., triple negative breast cancer (TNBC) [8]. Given the high prevalence of CA and its association with tumor aggressiveness, therapeutics targeting cancer cells with supernumerary centrosomes (described as “Achilles’ heel of cancer” [9]) represent a unique opportunity to eliminate the most aggressive tumors while sparing the normal cells.

Centrosomes are the major microtubule organizing centers within the cells. During mitosis, centrosomes aid in the formation and orientation of the mitotic spindles and support bipolar division. Clustering extra centrosomes into opposite spindle poles during mitosis is critical for preventing multipolar division and apoptosis in cancer cells with CA [10]. Along these lines, targeting members of the chromosomal passenger complex (CPC), e.g., Aurora A kinase and the kinetochore component, Ndc80 have previously been shown to prevent centrosome clustering (CC) and cause multipolar mitosis in centrosome-amplified cells in vitro [11, 12]. However, such mitosis-directed strategies have mostly been unsuccessful in clinical settings with poor efficacy [13] and severe side effects [14, 15]. It has yet to be determined whether these inhibitors will be effective in cancer patients with CA. Furthermore, identification of multifunctional CA-driven therapeutic targets that have key roles beyond mitosis is highly crucial to achieve durable anti-tumor effect with minimal toxicity.

Transforming acidic coiled-coil 3 (TACC3) is upregulated in solid tumors and strongly associated with worse prognosis in several different cancers, such as breast [16], lung [17] and ovarian cancer [18]. It is localized to centrosomes as well as microtubules and controls spindle stability and microtubule nucleation [19, 20]. Inhibiting TACC3 in in vitro settings has been shown to cause spindle defects and mitotic catastrophe [19]. Furthermore, we recently demonstrated that targeting TACC3 using a potent TACC3 inhibitor (BO-264) also has a strong anti-tumorigenic effect in vivo and it increases survival without any major toxicity [21]. Despite the growing body of evidence showing strong prognostic value of TACC3 in cancer and a therapeutic potential for targeting TACC3 to inhibit tumor growth, little is known about the molecular mechanisms of TACC3-driven tumor growth. Furthermore, whether TACC3 inhibition is an effective strategy to target highly aggressive tumors with CA has yet to be determined.

In this study, we identified TACC3 as being strongly upregulated in cancers with CA and associated with worse clinical outcome in highly aggressive patient subpopulations, especially in TNBC. We demonstrated that TACC3 is not only a key regulator of clustering centrosomes and mitotic progression, but also controls G1/S transition via forming distinct functional interactomes during cell cycle progression in cancer cells with CA. Notably, targeting TACC3 using genomic knockout or pharmacologic inhibition strongly inhibited tumor growth without any observable toxicity in cell line or patient-derived xenografts with CA.

## Results

### High TACC3 correlates with disease aggressiveness in patients with CA and breast cancer cells with CA are highly sensitive to TACC3 inhibition

**Fig. 1:**
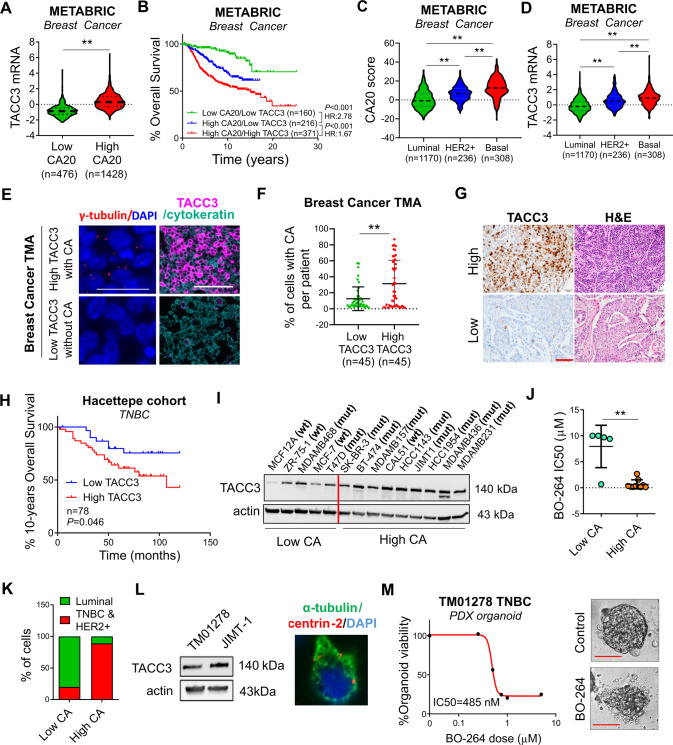
TACC3 correlates with disease aggressiveness in patients with CA and breast cancer cells/organoids with CA are highly sensitive to TACC3 inhibition. **A** TACC3 mRNA expression in breast cancer tumors in the METABRIC dataset with low vs. high CA20 score. **B** Survival analyses in breast cancer patients based on CA20 score and TACC3 expression. **C**, **D** Expression of CA20 score (**C**) and TACC3 mRNA (**D**) in different breast cancer subtypes in METABRIC dataset. **E** Representative IF images of breast tumors with or without CA (shown by γ-tubulin in red) with high vs. low TACC3 (magenta) expression from tissue microarray (BR1902, TissueArray). The epithelial marker, cytokeratin is shown in cyan and the nuclear marker, DAPI is shown in blue. Scale bar = 25 µm for γ-tubulin images and 50 µm for TACC3 + cytokeratin. **F** Percent of cells with CA per patient in low vs. high TACC3 protein-expressing tumors from tissue microarray (BR1902, TissueArray). **G** Immunohistochemistry staining of TACC3 and H&E staining in TNBC patients from Hacettepe cohort, showing representative low vs. high TACC3-expressers. Scale bar = 100 µm. **H** Kaplan Meier survival analysis showing 10-years overall survival in TNBC patients from Hacettepe cohort, separated based on median TACC3 protein expression. **I** TACC3 expression in a panel of breast cancer cell lines with CA and without CA. The red vertical line is added to show low vs. high CA cell lines. wt: p53 wild type, mut: p53 mutant. **J** Sensitivity of breast cancer cell lines from I to TACC3 inhibitor, BO-264 with respect to CA status. **K** Percentage of breast cancer cell lines from I belonging to TNBC and HER2 + subtype vs. luminal subtype based on CA status. **L** Western blot analysis of TACC3 in TNBC PDX TM01278 organoids in comparison with JIMT-1 cells (left panel) and α-tubulin (green) and centrin 2 (red) staining in TM01278 organoids showing CA (right panel). **M** Dose-response curve of TM01278 organoids upon BO-264 treatment for 1 week. The representative images are provided on the right panel. Scale bar = 100 µm. Actin is used as a loading control in all blots. CA Centrosome amplification, HR Hazard ratio.

To analyze the expression of TACC3 in breast cancer patients with CA, patients were stratified based on their CA status by using a published gene signature of CA (CA20). This list contains 20 genes that have been experimentally demonstrated to cause CA when dysregulated [22]. Importantly, TACC3 is not in this gene signature. As shown in Fig. 1A, TACC3 is higher in breast cancer tumors with high CA20 score in the METABRIC dataset [23], and among high CA20-expressing patients, those who express higher TACC3 exhibit much worse survival as compared to those who express lower TACC3 (Fig. 1B), suggesting that TACC3 may be critical for the outcome of patients with CA. Importantly, CA20 score and TACC3 levels were higher in HER2 + and basal subtypes of breast cancer, which are the two most aggressive subtypes (Fig. 1C, D). These results were further validated in an independent breast cancer dataset, GSE25066 (Supplementary Fig. S1A–D). To support the mRNA- and gene signature-based analysis for TACC3 and CA, respectively, we stained TACC3 protein in breast cancer patients of all subtypes (BR1902, TissueArray) along with the centrosome marker, γ-tubulin to assess CA (Fig. 1E). Importantly, we observed a significantly higher percent of cells with CA per patient in tumors with high TACC3 as compared to low TACC3 protein-expressing tumors from tissue microarray (BR1902, TissueArray) (Fig. 1E, F), supporting our findings using TACC3 mRNA expression and the mRNA-based scoring of CA. Furthermore, the percent of cells with CA per patient and TACC3 protein levels were higher in grade 3 tumors compared to grade 2 tumors **(**Supplementary Fig. S1E, F). Notably, in another patient cohort (Hacettepe cohort) which has follow up data, we demonstrated that high TACC3 protein expression is significantly associated with worse overall survival in TNBC subtype (Fig. 1G, H), further supporting the role of TACC3 in highly aggressive breast tumors with CA.

In a panel of breast cancer cell lines, we observed that the cancer cell lines with CA (mostly TNBC and HER2 + ) express higher levels of TACC3 and are more sensitive to TACC3 inhibition with BO-264 [21] (Fig. 1I–K). Furthermore, a TNBC PDX organoid expressing TACC3 and bearing CA (Fig. 1L) responded to BO-264 with a nanomolar range IC50 (Fig. 1M). Furthermore, TACC3 expression significantly correlates with CA20 score in the pan-cancer CCLE dataset, comprising the gene expression profiling of more than 1000 cell lines from 36 different cancer types (Supplementary Fig. S1G). Importantly, we showed that TACC3 is higher and it is associated with worse survival in CA tumors of not only breast cancer, but also of prostate (*P* = 0.001, HR = 7.25), lung (*P* = 0.049, HR = 2.19) and head & neck (*P* = 0.12, HR = 1.96) cancers (Supplementary Fig. S1H–M), suggesting that its association with CA is not restricted to breast cancer, but could be more general.

### TACC3 is associated with centrosome clustering (CC) in patients with CA and mediates CC in cancer cells with CA

**Fig. 2:**
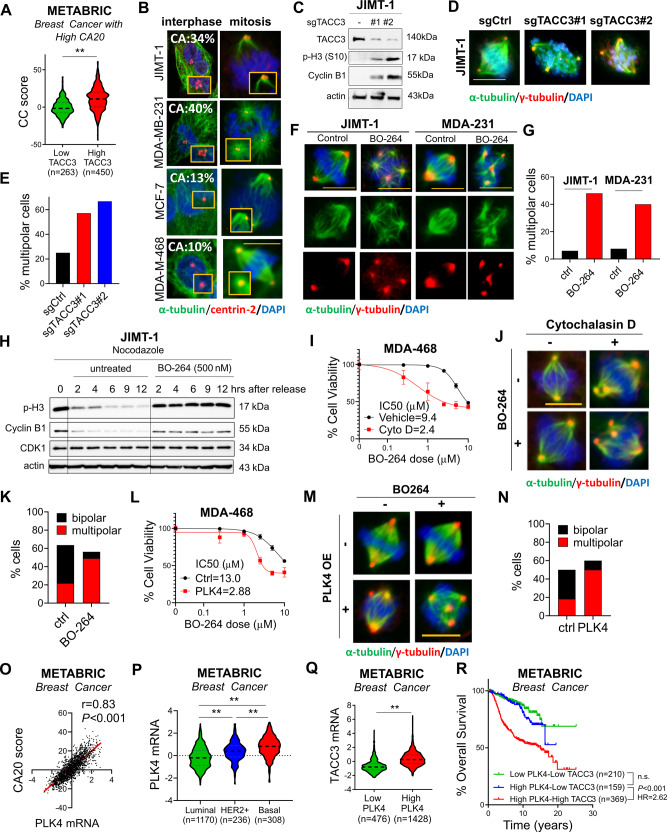
TACC3 correlates with centrosome clustering (CC) in patients with CA and mediates CC in cells with CA. **A** Expression of CC score in breast tumors with high CA20 expression from METABRIC with low vs. high TACC3 expression. **B** CA status (as % of cell population) of breast cancer cell lines determined by centrin-2 (red) and α-tubulin (green) in interphase and mitosis. Scale bar = 10 µm. **C** Western blot analysis of TACC3 and the mitotic arrest markers, p-H3 (S10) and Cyclin B1 in JIMT-1.sgCtrl vs. sgTACC3 cells. **D** Multipolar spindle formation in JIMT-1.sgCtrl vs. sgTACC3 cells as shown by α- (spindle, green) and γ- (centrosome, red) tubulin staining. Scale bar = 10 µm. **E** Quantification of mitotic cells with multipolar spindles from **D**. **F** Multipolar spindle formation in BO-264-treated JIMT-1 and MDA-MB-231 (MDA-231, here and for all figures) cells as shown by α- (spindle, green) and γ- (centrosome, red) tubulin staining. Scale bar=10 µm. **G** Quantification of mitotic cells with multipolar spindles from **F**. **H** Western blot analysis of mitosis markers in JIMT-1 cells synchronized at mitosis using nocodazole followed by release into fresh vs. BO-264-containing media. **I** Dose response curve of MDA-MB-468 (MDA-468, here and for all figures) cells 72 h after treatment with BO-264 upon CA induction with 1 µM of cytochalasin D for 20 h. **J** IF staining of α- (green) and γ- (red) tubulin in MDA-MB-468 cells treated with 1 µM of cytochalasin D for 20 h followed by 24 h treatment with 5 µM BO-264. Scale bar = 10 µm. **K** Quantification of mitotic cells with multipolar spindles from **J**. **L** BO-264 dose response curve of MDA-MB-468 cells transfected with control vector or PLK4 to induce CA. **M** IF staining of α- (green) and γ- (red) tubulin in MDA-MB-468 cells transfected with control or PLK4 vector for 24 h followed by treatment with 5 µM BO-264 for an additional 24 h. Scale bar = 10 µm. **N** Quantification of mitotic cells with multipolar spindles from **M**. **O** Correlation of PLK4 expression with CA20 score in breast cancer patients from METABRIC. **P** Expression of PLK4 in different breast cancer subtypes in METABRIC dataset. **Q** TACC3 mRNA expression in breast cancer tumors with low vs. high PLK4 expression. **R** Survival analyses in breast cancer patients based on PLK4 and TACC3 expression. CC centrosome clustering.

Centrosome clustering (CC) is a critical process required for bipolar division and survival of cancer cells with CA. To test if TACC3 has a role in CC, we examined the correlation of TACC3 with a CC-related gene signature [10] in breast cancer patients with CA. We observed that patients with high CA20 expression and high TACC3 expression and therefore have worse survival (as shown in Fig. 1B) exhibit higher levels of the CC score (Fig. 2A). These results were also validated in other tumors with CA, i.e., prostate, lung, and head & neck cancers (Supplementary Fig. S2A–C). Notably, TACC3 expression correlates with CC score in the pan-cancer CCLE dataset with CA20 expression (Supplementary Fig. S2D) and the NCI60 cell line panel (Supplementary Fig. S2E). Cancer cells having high CC scores from the NCI60 panel were shown to be more sensitive to TACC3 inhibitor, BO-264 (Supplementary Fig. S2F), further suggesting the critical involvement of TACC3 in mediating CC and cell survival.

To experimentally demonstrate the role of TACC3 in CC, we selected two breast cancer cell lines with CA (HER2 + cell line, JIMT-1 and TNBC cell line, MDA-MB-231), and two non-CA cell lines (luminal A cell line, MCF-7 and TNBC cell line, MDA-MB-468) as negative controls (Fig. 2B). We performed CRISPR Cas9-mediated knockout of TACC3 (named sgTACC3) in JIMT-1 cells with CA and MDA-MB-468 cell without CA (Fig. 2C, Supplementary Fig. S3A). While TACC3 knockout in JIMT-1 cells caused an increase in the percentage of multipolar cells with scattered centrosomes (Fig. 2D, E) and subsequently mitotic arrest (Fig. 2C), there was no increase in multipolar cell population with no mitotic arrest in MDA-MB-468 cells (Supplementary Fig. S3A–C). Furthermore, TACC3 inhibition using BO-264 caused multipolar mitosis only in JIMT-1 and MDA-MB-231 cells with CA **(**Fig. 2F, G), but not in the non-CA MCF-7 and MDA-MB-468 cells (Supplementary Fig. S3D, E). To demonstrate the direct effects of TACC3 on mitotic progression, potentially specific to cancer cells with CA, we synchronized JIMT-1 and MCF-7 cells at mitosis using nocodazole, followed by release into fresh or BO-264-containing media. While JIMT-1 cells released into BO-264-containing media strongly arrested at mitosis as shown by sustained cyclin B1 expression and p-H3 levels (Fig. 2H), MCF-7 cells released into BO-264-containing media could still progress through mitosis (Supplementary Fig. S3F), demonstrating the key role of TACC3 in mitotic progression, potentially specific to cancer cells with CA.

Induction of CA with the cytokinesis inhibitor, cytochalasin D [10, 24], in MDA-MB-468 cells resulted in an increased sensitivity (IC50 change from 9.4 µM to 2.4 µM) to BO-264 (Fig. 2I) by causing de-clustering of the extra centrosomes generated upon cytochalasin D treatment (Fig. 2J, K). Overexpression of PLK4, another known inducer of CA [25], induced CA in MDA-MB-468 cells, and TACC3 inhibition in these cells led to multipolar mitosis and higher growth inhibition (Fig. 2L–N), replicating the results with cytochalasin D. To demonstrate the clinical association of TACC3 with CA that is caused by PLK4 overexpression, we analyzed the METABRIC dataset [23] and showed that PLK4, whose expression correlates with CA20 score (Fig. 2O), is expressed at higher levels in HER2 + and basal subtypes (Fig. 2P). Patients with high PLK4 expression express higher TACC3 mRNA (Fig. 2Q) and have drastically worse overall survival (Fig. 2R), further validating the clinical relevance of TACC3 in cancers with CA.

### KIFC1 is a novel TACC3 binding protein involved in TACC3-mediated CC

**Fig. 3:**
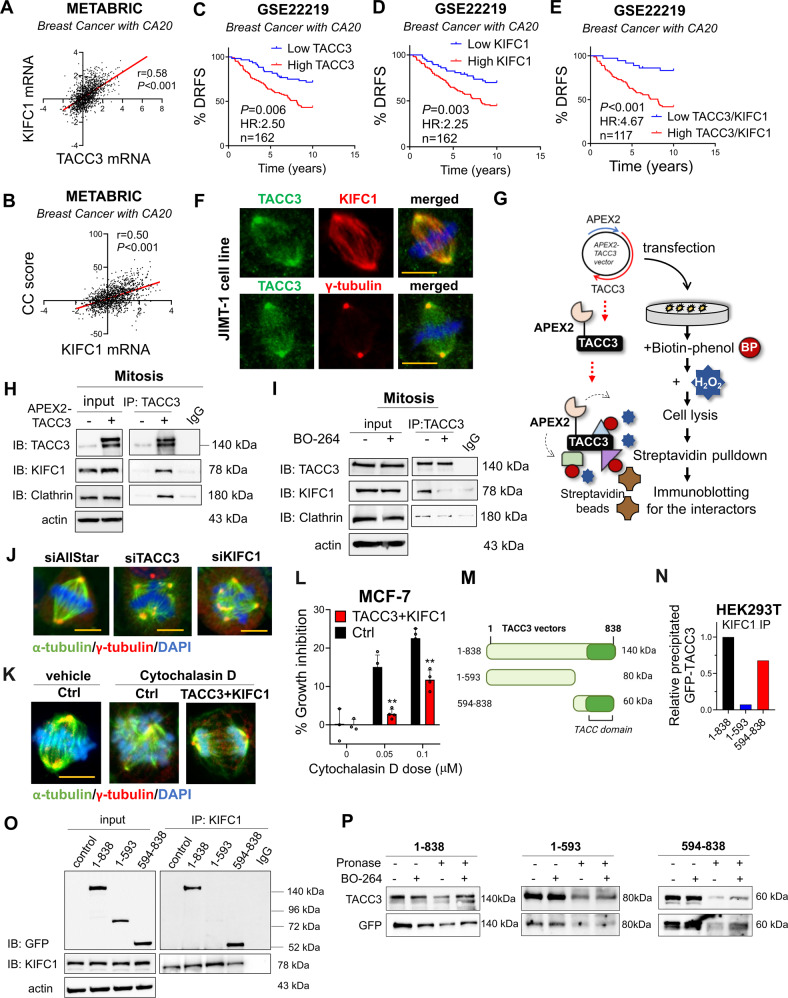
KIFC1 is a novel TACC3 binding protein that is involved in TACC3-mediated CC. **A**, **B** Correlation of KIFC1 mRNA expression with TACC3 (**A**) and CC score (**B**) in breast cancer patients with high CA20 expression in METABRIC dataset. **C**–**E** Distant relapse-free survival (DRFS) analyses in breast cancer patients with high CA20 expression based on TACC3 or KIFC1 or their combination in GSE22219 dataset. **F** IF staining of TACC3 (green)/KIFC1 or γ-tubulin (red) in JIMT-1 cells, showing colocalization at the centrosomes. Scale bar = 10 µm. **G** APEX2 proximity ligation assay to show binding of TACC3 to its interactors. **H** Western blotting of KIFC1 and the known interactor, clathrin upon TACC3 pulldown in APEX2-TACC3 overexpressing mitotic JIMT-1 cells. **I** IP of endogenous TACC3 and its interactors, KIFC1 and clathrin in JIMT-1 cells synchronized at mitosis and treated with BO-264 for 4 h (5 µM). **J** IF staining of α- (green) and γ- (red) tubulin in JIMT-1 cells transfected with siTACC3 or siKIFC1 (100 nM) for 48 h. Scale bar = 10 µm. **K** IF staining of α- (green) and γ- (red) tubulin in MCF-7 cells upon TACC3 + KIFC1 overexpression followed by CA induction with cytochalasin D. Scale bar = 10 µm. **L** Percent growth inhibition in MCF-7 cells upon TACC3/KIFC1 overexpression followed by CA induction with cytochalasin D. **M** A scheme of different truncated vectors of TACC3. **N** Quantification of the band intensities from **O**. **O** IP of KIFC1 and immunoblotting (IB) with GFP antibody in mitotic HEK293T cells transfected with GFP-labelled vectors of different regions of TACC3. 1-838: full length, 1-593: N-terminus, 594-838; C-terminus. **P** DARTS assay showing binding of BO-264 to the C-terminal TACC domain of TACC3.

Having demonstrated the phenotypic effects of TACC3 inhibition on CC, we next sought to identify the molecular mechanisms of TACC3-mediated CC in cancer cells with CA. The kinesin-14 family member, KIFC1 is among the few known CC-mediating proteins [26]. We first examined if there is a correlation between TACC3 and KIFC1 in clinical samples. We demonstrated that TACC3 expression strongly positively correlates with KIFC1 mRNA, which is also correlated well with the CC score in breast cancer patients with high CA20 expression in the METABRIC dataset (Fig. 3A, B). Notably, in these patients, combined expression of high TACC3 and high KIFC1 is more strongly associated with worse distant relapse-free survival (DRFS) compared to that of individual genes (Fig. 3C–E), suggesting that KIFC1 might work together with TACC3 in mediating CC in the CA cells.

To experimentally test this hypothesis, we first examined a potential interaction between the two proteins and showed co-localization of TACC3 and KIFC1 at the centrosomes of mitotic JIMT-1 cells by immunofluorescence staining (IF) (Fig. 3F). We further validated the binding between the two proteins in mitotic cells by a proximity ligation assay where we cloned TACC3 to the C-terminus of a modified peroxidase enzyme, APEX2 [27] (Fig. 3G). We confirmed the spindle and centrosome localization of APEX2-TACC3 in mitotic cells (Supplementary Fig. S4A). Immunoprecipitation (IP) of TACC3 in APEX2-TACC3 overexpressing mitotic JIMT-1 cells revealed a strong binding to KIFC1 (Fig. 3H) in addition to its known interactor, clathrin [28]. This is also verified upon H_2_O_2_ administration to induce specific biotinylation of the interactors, followed by streptavidin pulldown and immunoblotting for KIFC1 and ch-TOG, another known interactor of TACC3 [29] (Supplementary Fig. S4B, C). We further validated the binding between endogenous TACC3 and KIFC1 proteins by immunoprecipitating endogenous TACC3 in JIMT-1 cells (Fig. 3I). Importantly, the TACC3-KIFC1 interaction was abrogated upon BO-264 treatment, along with the known interactor, clathrin, as a potential molecular mechanism of TACC3 inhibition-mediated centrosome de-clustering (Fig. 3I). Supporting this, silencing KIFC1 with an siRNA phenocopied the effects of TACC3 knockdown in terms of the formation of multipolar spindles (Fig. 3J, Supplementary Fig. S4D, E). Furthermore, reconstituting TACC3 + KIFC1 complex in the non-CA MCF-7 cells treated by cytochalasin D to induce CA facilitated CC (Fig. 3K) and increased cell survival (Fig. 3L).

To identify the KIFC1-binding region on TACC3, we transfected HEK293T cells with the full length TACC3 (1-838 aa), N-terminal (1-593 aa) and C-terminal (594-838 aa) of TACC3. These vectors express GFP and also shTACC3 to silence endogenous TACC3 protein [30]. KIFC1 IP followed by immunoblotting with a GFP antibody revealed that the C-terminal region that corresponds to the TACC domain primarily interacts with KIFC1 (Fig. 3M–O). Importantly, the C-terminal region was also identified as the interacting region for the TACC3 inhibitor, BO-264 with DARTS (a drug-protein interaction assay [31]), partially explaining how BO-264 inhibits the TACC3/KIFC1 interaction (Fig. 3P, Supplementary S4F, G). Overall, these data demonstrate that (i) KIFC1 and TACC3 levels strongly correlate with each other as well as with the CC score and patient survival in the context of CA, (ii) KIFC1 is a novel interactor of TACC3 in mitotic cells, and (iii) the TACC3-KIFC1 complex plays a role in mediating CC and cell survival in cancer cells with CA.

### p53 loss/mutation increases the expression of TACC3 and KIFC1 via FOXM1 and renders cancer cells highly sensitive to TACC3 inhibition

**Fig. 4:**
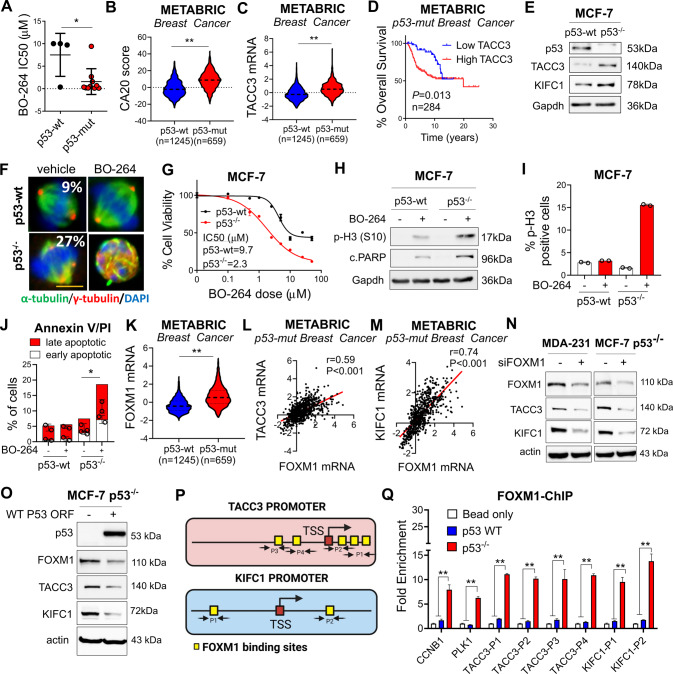
p53 loss/mutation increases the expression of TACC3 and KIFC1 via FOXM1 and renders cancer cells highly sensitive to TACC3 inhibition. **A** BO-264 IC50 values in breast cancer cell lines from Fig. 1I, separated based on their p53 mutational status. **B**, **C** CA20 score (**B**) and TACC3 (**C**) expression in p53-wt vs. p53-mut breast cancer patients in METABRIC dataset. **D** Survival analyses in p53-mut breast cancer patients based on TACC3 expression in METABRIC dataset. **E** Western blot analysis of p53, TACC3 and KIFC1 in MCF-7 p53-wt vs p53^−/−^ cells. **F** IF staining of α- (green) and γ- (red) tubulin in MCF-7 p53-wt vs p53^−/−^ cells treated with 5 µM of BO-264. Scale bar = 10 µm. **G** Dose response curve of MCF-7 p53-wt vs p53^−/−^ cells treated with BO-264 for 72 h. **H** Western blot analysis of the mitotic arrest marker, p-H3 (S10) and apoptosis marker, cleaved PARP in MCF-7 WT vs. p53^−/−^ cells treated with 2 µM of BO-264. **I**, **J** Flow cytometry analysis of p-H3 (**I**) and Annexin V/PI staining (**J**) in cells from **G**. **K** FOXM1 mRNA expression in p53-wt vs. p53-mut breast cancer patients from METABRIC. **L**, **M** Correlation of FOXM1 mRNA with TACC3 (**L**) and KIFC1 (**M**) expression in p53-mut breast cancer patients from METABRIC. **N** Western blot analysis of FOXM1, KIFC1 and TACC3 in siFOXM1-transfected MDA-MB-231 and MCF-7 p53^−/−^ cells. **O** Western blot analysis of p53, FOXM1, KIFC1 and TACC3 in MCF-7 p53^−/−^ cells transfected with wt p53 ORF. **P** A scheme showing the predicted binding FOXM1 sites (yellow squares) and regions targeted by primers (P1-P4 for TACC3 and P1-P2 for KIFC1) on TACC3 and KIFC1 promoters. TSS Transcription start site. **Q** FOXM1 ChIP assay in MCF-7 p53-wt and p53^−/−^ cells.

p53 alterations are among the major causes of CA in tumors [6]. We observed that cell lines with mutant p53 (mut-p53) exhibit CA (Fig. 1I) and are more sensitive to the TACC3 inhibitor, BO-264 (Fig. 4A). To test the clinical relevance of TACC3 in association with p53 mutations, we analyzed the METABRIC dataset by stratifying patients based on their p53 mutation status. Breast tumors with mut-p53 exhibit significantly higher levels of the CA20 score and TACC3 mRNAs (Fig. 4B, C). These results were further recapitulated in prostate, lung and head & neck tumors (Supplementary Fig. S5). Notably, TACC3 expression significantly correlates with CC score in mut-p53-bearing tumors (Supplementary Fig. S5C, F, I), and breast cancer patients with mut-p53 and high TACC3 expression demonstrate much worse overall survival as compared to patients with low TACC3 expression (Fig. 4D).

To experimentally test the effects of p53 loss on CA and determine the sensitivity to TACC3 inhibition in cancer cells with p53 loss or mutation, we first used p53 null (p53^−/−^) derivatives of the non-CA cell line, MCF-7 which was generated via CRISPR-Cas9 (Fig. 4E). Percentage of cells with CA increased to 27% from 9% upon p53 loss, validating the causal role of p53 in CA (Fig. 4F). TACC3 inhibition caused centrosome de-clustering, multipolar mitosis and a stronger reduction in cell viability in p53^−/−^ cells compared to p53-wt cells (Fig. 4F, G, Supplementary Fig. 6A). In line with this, we observed a stronger mitotic arrest and apoptosis induction in the p53^−/−^ cells compared to p53-wt counterparts upon TACC3 inhibition (Fig. 4H–J, Supplementary Fig. S6B).

Given the high dependence of the p53^−/−^ cells on TACC3 for CC and cell survival, we sought to analyze its expression together with its interaction partner KIFC1 in p53^−/−^ vs. wt MCF-7 cells. Intriguingly, the expressions of both TACC3 and KIFC1 are increased in p53^−/−^ cells compared to p53-wt cells at both protein and mRNA levels (Fig. 4E, Supplementary Fig. S6C). FOXM1 is a transcription factor that is repressed by p53 and responsible for transcription of mitosis-related genes [32]. Analysis of the METABRIC dataset revealed significant overexpression of FOXM1 in high CA20 expressing (Supplementary Fig. S7A) as well as in p53-mut breast tumors (Fig. 4K). A significant positive correlation between FOXM1 and TACC3/KIFC1 mRNAs in high CA20 expressing (Supplementary Fig. S7B, C) and in p53-mut patients (Fig. 4L, M) was observed, suggesting that FOXM1 could be a potential upstream regulator of TACC3 and KIFC1 in high CA, p53-mut tumors. Knocking down FOXM1 prominently reduced TACC3 and KIFC1 expressions in the p53-mut MDA-MB-231 and MCF-7 p53^−/−^ cells (Fig. 4N), demonstrating the upstream regulatory role of FOXM1. Importantly, overexpressing wt p53 in the p53^−/−^ MCF-7 cells reduced FOXM1 levels as well as the TACC3 and KIFC1 expressions (Fig. 4O). Furthermore, ChIP assay of FOXM1 showed that FOXM1 binds to the promoter regions of TACC3 and KIFC1 (in addition to its known targets, CCNB1 and PLK1 [33, 34]) in MCF-7 p53^−/−^ cells stronger than in p53-wt MCF-7 cells (Fig. 4P, Q). Overall, p53 loss/mutation increases the expression of TACC3 and KIFC1 via FOXM1 and renders cancer cells highly sensitive to TACC3 inhibition.

### TACC3 interacts with the members of the nucleosome remodeling and deacetylase (NuRD) complex in interphase cells with CA and inhibition of this interaction leads to G1 arrest and apoptosis

**Fig. 5:**
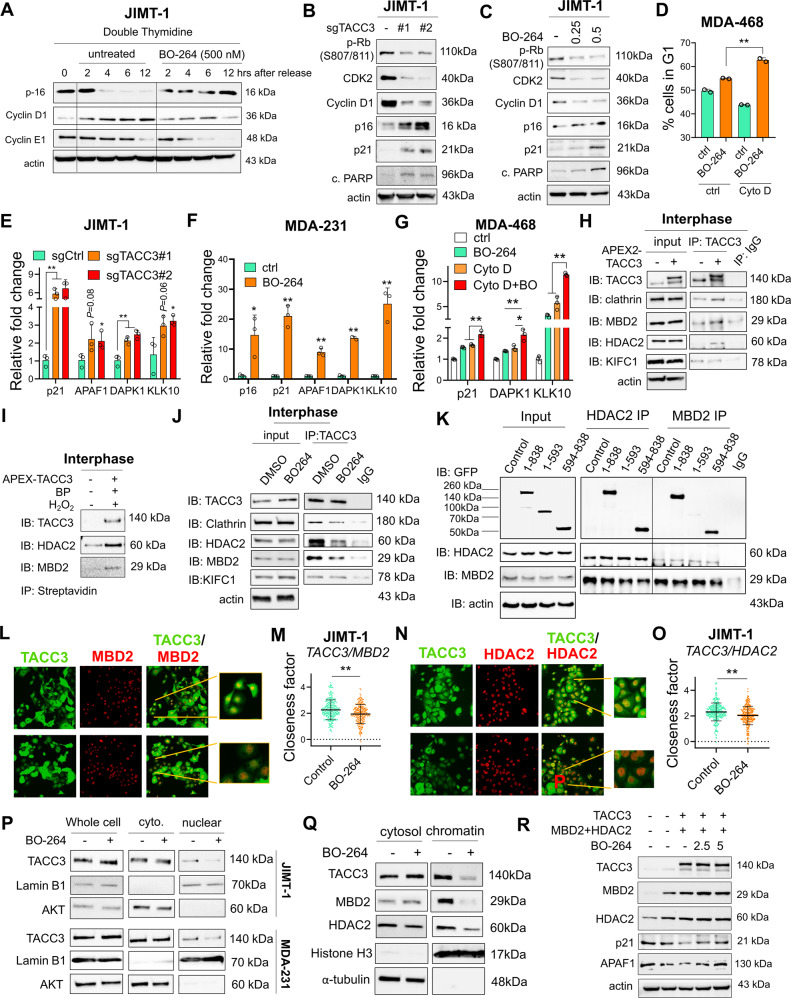
TACC3 interacts with the members of the nucleosome remodeling and deacetylase (NuRD) complex in interphase cells and inhibition of this interaction leads to G1 arrest and apoptosis. **A** Western blot analysis of G1/S progression markers and CDK inhibitor in JIMT-1 cells synchronized at G1 using double thymidine block followed by release into fresh vs. BO-264-containing media. **B** Western blot analysis of G1/S progression markers, CDK inhibitors and apoptosis in JIMT-1 sgCtrl and sgTACC3 cells. **C** Western blot analysis of G1/S progression markers, CDK inhibitors and apoptosis in JIMT-1 cells treated with 0.25 and 0.5 µM of BO-264. **D** Flow cytometry analysis with DAPI staining in MDA-MB-468 cells treated with 1 µM of cytochalasin D to induce CA followed by 24 h of 5 µM BO-264. **E** qRT-PCR analysis of NuRD complex targets in JIMT-1 sgTACC3 cells. **F** and **G** qRT-PCR analysis of NuRD complex targets in MDA-MB-231 cells (**F**) and MDA-MB-468 cells upon CA induction by cytochalasin D (**G**) under BO-264 treatment. **H** Western blot analysis of TACC3 interactors upon TACC3 pulldown in APEX2-TACC3 overexpressing interphase synchronized JIMT-1 cells. **I** Western blot analysis of TACC3 interactors upon biotinylation by H_2_O_2_ followed by streptavidin pulldown in interphase synchronized JIMT-1 cells. **J** IP of endogenous TACC3 and its interactors in JIMT-1 cells synchronized at interphase and treated with BO-264 (5 µM) for 4 h. **K** IP of HDAC2 or MBD2, and IB with GFP antibody in HEK293T cells transfected with GFP-labelled vectors of different regions of TACC3. 1-838: full length, 1-593: N-terminus, 594-838; C-terminus. **L**, **M** IF staining of TACC3 (green) and MBD2 (red) in JIMT-1 cells treated with BO-264 (**L**) and the closeness factor showing decrease in colocalization upon BO-264 treatment (**M**). **N**, **O** IF staining of TACC3 (green) and HDAC2 (red) in JIMT-1 cells treated with BO-264 (**N**) and the closeness factor showing decrease in colocalization upon BO-264 treatment (**O**). **P** Western blot analysis of TACC3 in cytoplasmic and nuclear fractions of interphase-synchronized JIMT-1 and MDA-MB-231 cells treated with 5 µM BO-264 for 6 h. Lamin B1 and AKT were used as nuclear and cytoplasmic markers, respectively. **Q** Western blot analysis of TACC3, and NuRD members in cytoplasmic and chromatin fractions of JIMT-1 cells treated with 5 µM BO-264 for 4 h. **R** Western blot analysis of TACC3, and NuRD members/targets in SK-BR-3 cells transfected with the overexpression vectors and treated with increasing doses of BO-264.

To test whether TACC3 may have novel functions beyond mitosis to promote cell cycle progression in cancer cells with CA, we synchronized JIMT-1 cells at G1 using double thymidine block, followed by release into fresh or BO-264 containing media. While cells released into fresh media progress through G1 to S phase, cells released into BO-264 containing media arrested at G1 (Fig. 5A). TACC3 knockout in unsynchronized JIMT-1 cells with CA has also led to a prominent increase in the CDK inhibitors, p21 (wt p53 target) and p16 and a decrease in G1/S progression markers, CDK2, cyclin D1 and RB phosphorylation that strongly correlate with the induction of apoptosis as shown by cleaved PARP (Fig. 5B). These data suggest that in addition to its roles in mitosis, TACC3 may also have crucial functions in G1/S progression that culminate in strong apoptosis in cancer cells with CA. These data were recapitulated by inhibiting TACC3 with BO-264 in two different CA cell lines with mutant p53 (Fig. 5C and Supplementary Fig. 8A). Notably, the induction of G1 arrest upon TACC3 inhibition was not observed in the non-CA MDA-MB-468.sgTACC3 cells (Supplementary Fig. S8B). On the other hand, when CA is induced in this non-CA MDA-MB-468 cells by treatment with cytochalasin D, cells underwent stronger G1 arrest upon TACC3 targeting (Fig. 5D), further suggesting a CA-driven dependence of cells to TACC3 for G1/S progression. Intriguingly, the G1 arrest-mediated apoptotic cell death under TACC3 inhibition (by sgRNAs or TACC3 inhibitor) was preceded by a strong induction of the mRNA levels of the CDK inhibitors and apoptosis inducers in CA or CA-induced cells (Fig. 5E–G), but not in non-CA cells (Supplementary Fig. S8C). These results suggest that TACC3 controls G1/S progression and cell survival via regulating the transcription of key tumor suppressors in interphase cells with CA in a p53-independent manner.

The nucleosome remodeling and deacetylase (NuRD) complex is one of the major chromatin remodeling complexes that plays important roles in processes, such as transcription, chromatin assembly, cell cycle progression and genomic stability [35, 36]. Intriguingly, APAF1, DAPK1 and KLK10, which are among the known targets of the NuRD complex [37, 38], regulating cell cycle progression and apoptosis along with p21 and p16 were also transcriptionally activated upon TACC3 knockout or BO-264 treatment in the CA cell line, MDA-MB-231 as well as in MDA-MB-468 cells upon CA induction with cytochalasin D (Fig. 5E–G). To identify the interphase-specific interactors of TACC3 that may be involved in TACC3-mediated transcriptional regulation and G1/S progression, we synchronized the APEX2-TACC3-overexpressing JIMT-1 cells at G1 using double thymidine block, followed by TACC3 or streptavidin IP. We validated the nuclear localization of APEX2-TACC3 in interphase cells via IF (Supplementary Fig. S8D). We identified one of the members of the NuRD complex, HDAC2 as a novel interactor of TACC3, along with the known interactor, MBD2 [39] in interphase cells with CA (Fig. 5H, I, Supplementary Fig. S8E). Furthermore, we demonstrated the interaction between endogenous TACC3, MBD2 and HDAC2 in G1-synchronized cells which is reduced upon short-term treatment with TACC3 inhibitor (Fig. 5J). These data correlate with the continued G1 arrest observed in G1-synchronized cells upon release into BO-264-containing media (Fig. 5A). Overexpressing the full-length, C-terminal and N-terminal regions of TACC3, followed by pull down of HDAC2 and MBD2 in HEK293T cells demonstrated a strong binding of the two NuRD complex members to TACC3 C-terminal region, bearing the TACC domain (Fig. 5K). Notably, inhibiting HDAC2 or MBD2 with siRNAs in JIMT-1 cells with CA partially recapitulated the effects of TACC3 inhibition on the expression of G1/S transition markers without causing mitotic arrest (Supplementary Fig. S8F). Likewise, we observed weak interaction of KIFC1 with TACC3 in interphase cells with no reduction upon TACC3 inhibition (Fig. 5H, J).

To elucidate the mechanisms of how TACC3 inhibition increases the transcription of tumor suppressors which are the targets of the NuRD complex, we first examined the co-localization of TACC3 with MBD2 and HDAC2 with or without TACC3 inhibition. We demonstrated co-localization of TACC3 with MBD2 and HDAC2 within the nucleus of CA cells by IF staining which was significantly reduced upon BO-264 treatment (Fig. 5L–O). The nuclear localization of TACC3 was further validated by fractionation assay in interphase of two different CA cell lines, JIMT-1 and MDA-MB-231 (Fig. 5P). Moreover, TACC3 inhibitor, BO-264 reduced the nuclear (Fig. 5P) as well as the chromatin-bound levels of TACC3 (Fig. 5Q) that could explain the decrease in TACC3 interaction with MBD2 and HDAC2 upon TACC3 inhibition. This suggests that loss of TACC3 interaction with the NuRD complex, and thus the transcriptional activation of tumor suppressors is involved in G1 arrest-mediated apoptosis under TACC3 inhibition in cancer cells with CA. Indeed, when TACC3 is overexpressed along with the NuRD complex members in the low TACC3/MBD2/HDAC2 expressing p53 mutant SK-BR-3 cells, the expression of the tumor suppressor NuRD target genes, p21 and APAF1 was reduced, while TACC3 inhibition with BO-264 restored their expression (Fig. 5R). Overall, these data suggest TACC3 interacts with the members of the NuRD complex in interphase cells with CA, and inhibition of this interaction leads to G1 arrest and apoptosis.

### Targeting TACC3 inhibits the growth of centrosome-amplified breast tumors

**Fig. 6:**
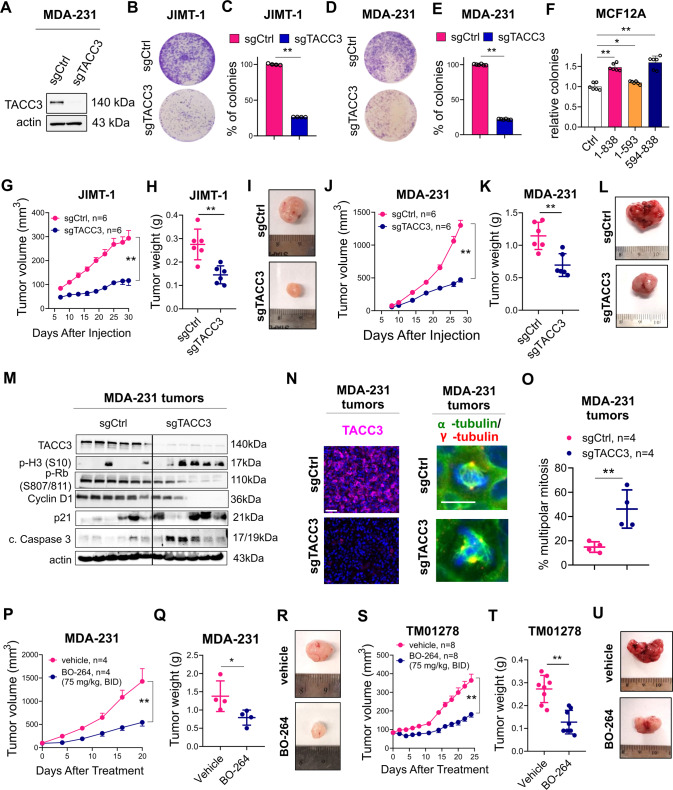
Targeting TACC3 inhibits tumor growth in centrosome-amplified breast tumors in vivo. **A** Western blot validation of CRISPR/Cas9-mediated knockout of TACC3 in MDA-MB-231 cells. **B–E** The effect of TACC3 knockout on colony formation in JIMT-1 (**B**, **C**) and MDA-MB-231 (**D**, **E**) cells. **F** Relative colony formation ability of MCF12A cells overexpressing different regions of TACC3. 1-838: full length, 1-593: N-terminus, 594-838; C-terminus. **G–I** Tumor growth **(G**) in xenografts of JIMT-1 sgCtrl vs. sgTACC3 cells, and the tumor weights and representative images at the end of the experiment (**H**, **I**). **J**–**L** Tumor growth (**J**) in xenografts of MDA-MB-231 sgCtrl vs. sgTACC3 cells, and the tumor weights and representative images at the end of the experiment (**K**, **L**). **M** Western blot analysis of TACC3, mitotic progression and G1/S progression markers, CDK inhibitor and apoptosis in MDA-MB-231 xenografts from **J**. **N** IF staining of TACC3 (magenta), α- (green) and γ- (red) tubulin in MDA-MB-231 xenografts from **J**. Scale bar = 40 µm for TACC3 and 20 µm α- and γ-tubulin. **O** Quantification of multipolar mitosis in MDA-MB-231 xenografts from **J**. **P** Tumor growth in xenografts of MDA-MB-231 cells treated with 75 mg/kg BO-264, twice daily (p.o.). **Q**, **R** The tumor weights (**Q**) and representative images (**R**) from vehicle vs. BO-264 treated mice from **P** at the end of the experiment. **S** Tumor growth of TNBC PDXs, TM01278 treated with 75 mg/kg BO-264, twice daily (p.o.). **T**, **U** The tumor weights (**T**) and representative images (**U**) from vehicle vs. BO-264 treated mice from **S** in the end of the experiment.

To test the effects of TACC3 inhibition on the growth of tumors with CA in vivo, we performed CRISPR Cas9-mediated knockout of TACC3 in MDA-MB-231 cells in addition to JIMT-1 cells (Fig. 2C) and validated the TACC3 depletion by Western blotting (Fig. 6A). TACC3 knockout significantly decreased the colony formation ability of the cells (Fig. 6B–E). To test whether the TACC domain of TACC3, which we found to be important for binding to KIFC1 and MBD2/HDAC2, is able to increase the transforming ability of normal cells, we transfected the normal breast cells, MCF12A with full length, N- and C-terminal domains of TACC3. As shown in Fig. 6F, the TACC domain (594-838) was sufficient to induce colony formation to a similar extent to full length, whereas the N-terminal region (1-593) failed to increase the transforming ability.

To test the effects of TACC3 knockout on in vivo tumor growth, we injected sgCtrl vs. sgTACC3-expressing JIMT-1 and MDA-MB-231 cells to the mammary fat pad (MFP) of nude mice and monitored tumor growth. As shown in Fig. 6G–L, TACC3 knockout reduced the growth of tumors with CA. The knockout of TACC3 in tumors was validated at protein level by both Western blot and IF staining in the end of the in vivo experiment (Fig. 6M, N). Importantly, the number of multipolar spindles was higher in TACC3-depleted tumors (Fig. 6N, O), resulting in mitotic arrest and apoptosis as shown by increased p-Histone H3 (S10) and cleaved caspase 3 (Fig. 6M). The G1/S progression markers, p-RB, and cyclin D1 were also strongly downregulated in TACC3 knockout tumors (Fig. 6M). The molecular alterations observed upon TACC3 knockout in CA tumors in vivo were also recapitulated upon short-term induction of shRNA-mediated TACC3 knockdown or inhibition with BO-264 in JIMT-1 xenografts (Supplementary Fig. S9A, B). In addition, we tested the effects of pharmacological inhibition of TACC3 on tumor growth in both MDA-MB-231 xenografts and the TNBC PDX, TM01278 with CA using the TACC3 inhibitor, BO-264. TACC3 inhibition significantly reduced the growth of the tumors without any observable toxicity as shown by the body weight changes of mice and the blood cell counts (Fig. 6P–U, Supplementary Fig. S10A–C). Notably, TACC3 inhibition with BO-264 did not reduce the growth of tumors of the non-CA MDA-MB-468 xenografts without any significant body weight loss (Supplementary Fig. S10D–G) and without a change in mitosis or G1/ progression (Supplementary Fig. S10H). Altogether, these data suggest that the identified mechanisms of TACC3 inhibition-mediated cell death in vitro are also relevant in vivo and TACC3 inhibition represents a novel vulnerability in tumors with CA.

## Discussion

**Fig. 7:**
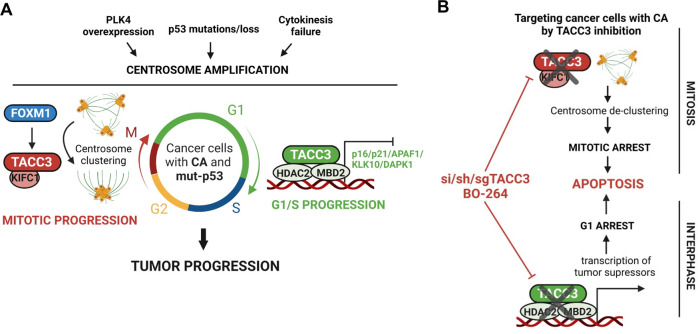
Schematic summary of the findings. **A** In cancer cells with CA that can be induced upon PLK4 overexpression, p53 modulation or cytokinesis failure, TACC3 is overexpressed and mediates distinct mitosis and interphase-specific functions to promote cell cycle and tumor progression. Mitotic TACC3 interacts with KIFC1 at the centrosomes and promotes CC to ensure mitotic progression and inhibition of apoptosis. FOXM1 mediates the transcription of TACC3 and KIFC1 during mitosis in p53^−/−^ or mutant cells. Interphase TACC3 interacts with MBD2 and HDAC2, belonging to the NuRD complex, to suppress the transcription of tumor suppressors (i.e., p16, p21, APAF1, KLK10 and DAPK1) and ensures G1/S transition. **B** Upon TACC3 targeting by a TACC3 inhibitor, BO-264 or si/sh/sgRNAs in cancer cells with CA, while loss of TACC3/KIFC1 interaction at the centrosomes of mitotic cells leads to centrosome de-clustering and mitotic cell death, loss of TACC3/HDAC2/MBD2 interaction at the nucleus of G1 cells leads to transcriptional activation of tumor suppressors to cause G1 arrest and apoptosis that overall culminates in inhibition of tumor growth.

CA is one of the hallmarks of cancer [1, 2] and is associated with tumor aggressiveness and worse clinical outcome in many cancers, including breast, lung and prostate cancers [2]. Targeting CC, which is required for faithful mitosis of cancer cells with CA, has been considered an optimal therapeutic approach to target tumors with CA. However, there is currently no therapeutic strategy that has yet reached clinics. Furthermore, little is known about the processes and players triggering aggressiveness of cancer cells with CA beyond mitosis. Here, we demonstrated, for the first time, that TACC3 is a novel CA-directed dependency that is overexpressed at mRNA and protein levels in high CA tumors, as determined by both mRNA-based scoring and protein-based immunostaining approaches and associated with worse clinical outcome in cancer patients with CA. Mechanistically, we found that TACC3 interacts with KIFC1, both of which are upregulated by FOXM1 upon p53 loss/mutation (an important inducer of CA), via its TACC domain in mitotic cells with CA to promote CC and facilitate mitotic progression. On the other hand, TACC3 interacts with the members of the nucleosome remodeling and deacetylase (NuRD) complex (HDAC2 and MBD2) in the nucleus of interphase cells with CA via its TACC domain, thereby suppressing the transcription of key tumor suppressors (e.g., p21, p16 and APAF1) to facilitate G1/S progression and cell survival (Fig. 7A). We demonstrated that inhibiting TACC3 not only blocks mitotic progression but also inhibits G1/S progression via disruption of the TACC3/KIFC1 complex in mitosis and the TACC3/HDAC2/MBD2 complex in interphase, respectively (Fig. 7B). Overall, our findings provide valuable preclinical data for targeting TACC3 in highly aggressive tumors bearing amplified centrosomes.

TACC3 localizes to spindles and centrosomes, regulates spindle stability and centrosome integrity and is required for proper cell division [19, 20]. It is phosphorylated by Aurora A during mitosis which is required for its spindle localization [40]. Aurora A is known to cause CA partly via phosphorylating p53 at S315, marking it for degradation [41]. Loss of p53 can, in turn, trigger CA via controling centrosome duplication [42]. Based on our novel findings that showed strong upregulation of TACC3 in p53^−/−^ or p53-mut cells/tumors, an interesting hypothesis to test would be whether Aurora A induces CA by regulating TACC3, or whether TACC3 has a novel, direct role in CA, independent of Aurora A. Furthermore, it may also be interesting to test whether TACC3 phosphorylation by Aurora A has any role in TACC3-mediated CC. We demonstrated that TACC3 promotes CC in mitotic CA cells via interacting with KIFC1 at its TACC domain using multiple approaches. KIFC1 is a kinesin motor protein which is known to induce spindle pole focusing [43]. Recently, the ATM/ATR kinases have been shown to promote CC under DNA damaging agents via phosphorylating KIFC1, leading to drug resistance [44]. In this line, it is yet to be determined whether KIFC1 phosphorylation may also be critical for the formation of the TACC3/KIFC1 complex in mitotic CA cells. Furthermore, unbiased proteomic approaches can identify the unknown members of the TACC3 interactome on centrosomes, and may unleash new drug targets regulating CC in the highly aggressive tumors with CA.

TACC3 has been shown to co-localize to nuclear envelope and maintain proper nuclear envelope structure [45]. It can also interact with MBD2 [39], a methyl-CpG-binding protein and a member of the NuRD complex, which regulate processes, such as transcription and chromatin assembly [35]. However, the functional consequences of these interactions in terms of gene expression and G1/S progression have not yet been studied. Here, we uncovered, for the first time, the interphase interactome of TACC3 in the nucleus of cancer cells with CA which we further demonstrated to be crucial for gene transcription and cell cycle progression. We identified HDAC2, a histone deacetylating enzyme that was shown to be a part of the MBD2-containing NuRD complex [36] as a novel interactor of TACC3, binding to C-terminal TACC domain in interphase cells with CA. We showed that TACC3 inhibition reduces nuclear TACC3 levels, decreases the levels of NuRD complex members (i.e., MBD2 and HDAC2) on the chromatin, and thus, relieving the inhibitory effect on the transcription of key tumor suppressors, i.e., p16, p21, APAF1, DAPK1 and KLK10 in a p53-independent manner, ultimately leading to G1 arrest and apoptosis. Among those, p21 and APAF1 are known to be direct targets of p53 [46, 47], suggesting that blocking NuRD complex via TACC3 inhibition can activate the transcription of tumor suppressors even in the absence of functional p53, e.g., in tumors with CA. Importantly, despite the key roles of the NuRD complex in tumor progression, there are no inhibitors specifically targeting NuRD complex. Based on our novel findings, TACC3 inhibition may represent a novel way of inhibiting NuRD complex in cancer.

A few mitotic proteins have been previously identified as potential targets for eliminating chromosomally instable (CIN) cancer cells. For instance, the mitotic kinesin, KIF18A has been shown to play a key role in maintaining bipolar spindle integrity and to be crucial for the survival of cancer cells with CIN [48]. Inhibitors against KIFC1 (e.g. AZ82 [49]) have been shown to be effective in vitro; however, their preclinical testing in cancer models is lacking. Inhibitors against Aurora kinases have already been clinically tested in solid tumors and hematologic malignancies; however, only poor or modest efficacy was observed, along with side-effects, such as neutropenia [15]. Alisertib (MLN8237) is the only Aurora A inhibitor progressed to Phase III evaluation in Peripheral T-Cell Lymphoma; however, no superior benefit was achieved over its investigator-selected single-agent comparator [50]. Therefore, it is of utmost importance to identify more effective therapeutic strategies against cancers with CA which preferentially target not only mitotic, but also non-mitotic cancer cells in tumors with heterogenous cell population to achieve superior and durable clinical efficacy [13]. Along these lines, TACC3 may represent an excellent drug target with a strong translational potential in the treatment of highly aggressive cancers as 1.) it is overexpressed in CA tumors compared to non-CA tumors and normal tissue; 2.) its inhibition blocks cell cycle progression at both mitosis and interphase with distinct mechanisms, assuring effective inhibition of cell proliferation; and 3.) its inhibition has strong anti-tumor activity with no apparent toxicity in vivo.

We demonstrated the CA-directed vulnerability to targeting TACC3 by inducing CA in non-CA cells using different approaches, such as PLK4 overexpression or cytokinesis inhibition by cytochalasin D which conferred high sensitivity to TACC3 inhibition. Percentage of tumors with CA is profoundly high in many different cancer types, such as invasive breast carcinoma and squamous cell carcinomas of the head and neck where percentage of tumors with hyper-amplified centrosomes goes up to 80% [51]. CA is also associated with several hallmarks of cancer related to tumor aggressiveness, such as aneuploidy that is observed in the vast majority (~70%) of solid tumors [52], and p53 mutations that occurs in ~50-60% of tumors, and can be seen in as high as 80% of patients of aggressive cancer subtypes, such as TNBCs [53]. Therefore, strategies targeting CA-driven vulnerabilities have the potential to be highly effective in aggressive tumor types where the percentage of cells with CA or the associated phenotypes is extremely high. Importantly, p53 mutations as well as amplified centrosomes were also shown to positively associate with tumor aggressiveness and development of metastatic disease [54]. Considering the roles of centrosomes in directing cell polarity and movement, it is highly likely that TACC3 may also be involved in tumor cell dissemination in the context of CA and p53 mutant cancers. Along these lines, targeting cancer cells with mut-p53 and/or CA by TACC3 inhibition would be a highly effective strategy against metastatic dissemination and would further improve clinical outcome at the later stages of the disease.

Overall, we identified TACC3 as a novel CC-mediator and a transcription-regulator overexpressed in highly aggressive tumors with CA and associated with drastically worse clinical outcome. We showed, for the first time, that TACC3 forms distinct functional interactomes during cell cycle progression (both mitosis and interphase) that are essential for the proliferation and survival of cancer cells with CA. These preclinical findings as well as the supporting clinical data strongly encourage the clinical testing of TACC3 inhibitors to improve the outcome of highly aggressive cancer patients bearing amplified centrosomes.

## Materials and Methods

### Cell culture and reagents

Human breast cancer cell lines, ZR-75-1, MDA-MB-231, MDA-MB-157, MDA-MB-436, MDA-MB-468, CAL51, HCC1954, JIMT-1, MCF-7, T47D, SK-BR-3, BT-474 and HCC1143 and the normal breast cells, MCF12A were obtained from ATCC (Manassas, VA, USA). All the cells were cultured in Dulbecco Modified Eagle Medium (Corning, NY, USA) supplemented with 50 U/ml penicillin/streptomycin, 1% non-essential amino acids and 10% fetal bovine serum (Corning, NY, USA). The media for ER + cell lines were further supplemented with insulin (0.1 µg/ml). The cell lines were authenticated and tested for mycoplasma contamination regularly using MycoAlert mycoplasma detection kit (Sigma, MA, USA). The cumulative culture length of cells between thawing and use in this study was less than 20 passages.

### Breast cancer tumor samples

To analyze the association of TACC3 protein with CA in breast tumors, a breast cancer tissue array (BR1902) was purchased from TissueArray, LLC. To analyze the association of TACC3 protein expression with clinical outcome in TNBC patients, we performed immunohistochemistry (IHC) staining of TACC3 in primary tumor samples from 78 TNBC patients that were diagnosed between 2000 and 2016 at Hacettepe University School of Medicine, Ankara, Turkey. The study was approved by the Non‐Interventional Clinical Research Ethics Committee of Hacettepe University (approval no: 2020/02-40). Informed consent was obtained from all patients.

### CRISPR/Cas9-mediated knockout studies

The sgRNA sequences targeting TACC3 in JIMT-1, MDA-MB-231 and MDA-MB-468 cells are 5’-CAGGCAACGTACCCTCAGCG-3’, and 5’-GACTTGGTGTCACCTCCGAA-3’. sgRNAs were designed and selected based on having high on-target (=high efficacy) and low off-target (=high specificity) activity using the CRISPick tool (Broad Institute). The designed sgRNAs were cloned into human lentiCRISPR v2 vector (Addgene, MA, USA). For lentiviral packaging, HEK293T cells were transfected with sgRNAs and the packaging plasmids, pMD2.G and psPAX2 (Addgene, MA, USA). Transduction of JIMT-1, MDA-MB-468 and MDA-MB-231 cells was performed in the presence of 10 ug/ml polybrene, and selection of transduced cells was done using 2 µg/ml puromycin.

### In vivo studies

Six-to-eight-week-old female BALB/c nude or Nu/J mice were housed with a temperature-controled and 12 h light/12 h dark cycle environment. All the in vivo studies were carried out in accordance with the Institutional Animal Care and Use Committee of the University of South Carolina and Medical University of South Carolina. The sgCtrl and sgTACC3-expressing derivatives of MDA-MB-231 and JIMT-1 cells were injected into MFPs of female BALB/c nude or Nu/J mice at a cell number of 5 × 10^6^ and 4 × 10^6^ cells, respectively, in 100 μl of 1:1 PBS and Matrigel (Corning, NY, USA), v/v) (6 mice per group). Primary tumor growth was monitored by measuring the tumor volume at least twice a week with a digital caliper. Tumor volumes were calculated as length × width^2^/2. All mice used were female and of the same age and similar body weight.

For testing the effects of TACC3 inhibitor, BO-264 on tumor growth, 5 × 10^6^ MDA-MB-231 cells or 10 × 10^6^ MDA-MB-468 cells were injected into MFPs of female BALB/c nude mice in 100 μl of 1:1 PBS and Matrigel. Once the mean and median of tumor volume reach around 100 mm^3^, xenografts were randomized into two groups (4 mice per group for MDA-MB-231 and 6 mice per group for MDA-MB-468). Animals were treated with vehicle or BO-264 (twice a day with 75 mg/kg oral gavage (po.)). The PDX experiment was carried out as previously described [55]. Briefly, 2 × 2 × 2 mm to 3 × 3 × 3 mm sized fragments were transplanted to the flank region of female Nu/J mice. Once the mean and median of tumor volume had reached around 85 mm^3^, PDXs were randomized into two groups (8 mice per group), and treated with vehicle or BO-264 (twice a day with 75 mg/kg, po.). For the short-term treatment of BO-264, 5 × 10^6^ JIMT-1 cells or 10 × 10^6^ MDA-MB-468 cells were injected into MFPs of Nu/J mice (4 mice per group for JIMT-1 and 5 mice per group for MDA-MB-468) and when average tumor volume is at 250 mm^3^, mice were treated with 50 mg/kg BO-264 and mice were sacrificed, and tumors were collected after 12 h. For the short-term induction of shTACC3, shTACC3 expressing JIMT-1 cells [21] (4 mice per group) were injected into MFP of Nu/J mice. When tumors reach 100 mm^3^, shRNA was induced by doxycycline treatment in drinking water (1 mg/kg) for 5 days. Mice were sacrificed and tumors were collected. There was no blinding during in vivo experiments. Sample sizes were determined based on previous studies [21, 55].

### Bioinformatics and statistical analyses

The METABRIC data [23] was downloaded from EMBL European Genome–Phenome Archive (http://www.ebi.ac.uk/ega/) with an accession number EGAS00000000122. The Cancer Genome Atlas (TCGA) [56] patient data (prostate cancer) and the CCLE data [57] were downloaded using the cBIO dataset [58]. The microarray data sets, GSE25066 [59], GSE22219 [60], GSE31210 [61] and GSE41613 [62] were download from the GEO database. The CA20 [22] and CC [10] signature scores were calculated by summing up the z-scores of the genes found in the list [63] using the SPSS Statistics software. Seventy five percent of breast cancer patients [64] and 83% of head & neck cancer patients [51], 50% of lung [65] and prostate [66] cancer patients were stratified as high CA based on the CA20 score expression. Cancer patients except breast cancer were separated as low vs. high TACC3 expressers based on median mRNA expression, and breast cancer patients were separated as low vs high TACC3 based on median or 25^th^ percentile mRNA expression. For the Hacettepe cohort, patients were separated based on median TACC3 protein expression as low TACC3 expressers (intensity = 0–25) vs. high TACC3 expressers (intensity = 30–210).

The results are represented as mean ± standard deviation (SD) or mean ± standard error of the mean (SEM), as indicated in the figure legends. All statistical analyses were performed in GraphPad Prism Software. Comparisons between two groups were done using paired two-sided Student’s t-test for tumor growth graphs, and unpaired two-sided Student’s t-test for all other comparisons. z-score calculations were done using the SPSS Statistics software. Survival curves were generated based on median or 25^th^ percentile separation using Kaplan-Meier method, and significance between groups was calculated by Log-rank test. For correlation analysis, Pearson correlation coefficients were calculated. Experiments were repeated two to three times independently with similar results.

The other methods, including inhibitor treatment, cell viability, transfection, PDX organoids, APEX2 proximity labeling, colony formation, qRT-PCR, Western blotting, DARTS, IP, chromatin fractionation, ChIP, IF and quantification, Annexin V and and cell cycle assays are provided in Supplementary Materials and Methods. The uncropped Western blots are provided in Supplementary Materials.

### Reporting summary

Further information on research design is available in the Nature Research Reporting Summary linked to this article.

## Supplementary information

Supplementary Information

Source Data for Western Blotting

Reporting Summary

## Data Availability

Gene expression data were downloaded from the NCBI Gene Expression Omnibus database under GSE31210, GSE22219, GSE25066, and GSE41613. METBARIC data were downloaded from EMBL European Genome–Phenome Archive (http://www.ebi.ac.uk/ega/) with an accession number EGAS00000000122. The Cancer Genome Atlas (TCGA) patient data (prostate cancer) and the CCLE data were downloaded using the cBIO dataset. The raw data for western blotting are provided as a source data file.
