# Supplementary material for: Targeting TACC3 represents a novel vulnerability in highly aggressive breast cancers with centrosome amplification
Source: Cell Death Differ. 2023 Mar 2;30(5):1305–19. doi: 10.1038/s41418-023-01140-1 (PMC10154422; doi:10.1038/s41418-023-01140-1)
Supplement: Supplementary file 1 — Supplementary Information [file 41418_2023_1140_MOESM1_ESM.docx]

**SUPPLEMENTARY INFORMATION**

**Targeting** **TACC3 represents a novel vulnerability in highly aggressive breast cancers with centrosome amplification**

Ozge Saatci^1,2^, Ozge Akbulut^1^, Metin Cetin^1,2^, Vitali Sikirzhytski^1^, Meral Uner^3^, Deniz Lengerli^4^, Elizabeth C. O’Quinn^5^, Martin J. Romeo^5^, Burcu Caliskan^4^, Erden Banoglu^4^, Sercan Aksoy^6^, Aysegul Uner^3^, Ozgur Sahin^1,2*^

^1^Department of Drug Discovery and Biomedical Sciences, University of South Carolina, Columbia, SC 29208, USA

^2^Department of Biochemistry and Molecular Biology, Hollings Cancer Center, Medical University of South Carolina, Charleston, SC 29425, USA

^3^Department of Pathology, Faculty of Medicine, Hacettepe University, 06100, Ankara, TURKEY

^4^Department of Pharmaceutical Chemistry, Faculty of Pharmacy, Gazi University, 06100, Ankara, TURKEY

^5^Translational Science Laboratory, Hollings Cancer Center, Medical University of South Carolina, Charleston, SC 29425, USA

^6^Department of Medical Oncology, Hacettepe University Cancer Institute, 06100, Ankara, TURKEY

**Running Title:** Targeting TACC3 in centrosome amplified breast cancer

**Keywords:** Centrosome amplification/centrosome clustering/TACC3/KIFC1/NuRD complex

**^*^Corresponding author**

Ozgur Sahin, PhD

Professor and SmartState Endowed Chair

Department of Biochemistry and Molecular Biology

Hollings Cancer Center

Medical University of South Carolina

86 Jonathan Lucas Street, Room HO712F, Charleston, SC 29425

Phone: +1-843-792-0166

E-mail: [sahinozgur@gmail.com](mailto:sahinozgur@gmail.com) or [sahin@musc.edu](mailto:sahin@musc.edu)

**Supplementary Materials and Methods**

**Inhibitor treatments and cell viability assays**

BO-264 and cytochalasin D, were dissolved in DMSO. For cell viability assays, cells were treated with the inhibitors for 48-72 hr, and cell viability was measured by Sulforhodamine B (SRB) (Sigma Aldrich). Induction of CA was performed by treating cells with 1 µM of cytochalasin D for 20 hr, followed by 24 hr incubation in drug-free media. The mitosis and interphase synchronizations were done using 100 ng/mL nocodazole for and double thymidine block (2 mM), respectively.

**PDX-derived organoids**

TNBC PDX organoids were established as previously described ^1^. For drug testing studies, organoids were dissociated with Tryple (Gibco) at 37 ºC for 30 min in the presence of 10µM Rock inhibitor (Selleckchem). After counting the single cells, they were plated into 96-well plate (20,000 cells/well) on a matrigel-coated surface with media containing 2% matrigel (Corning). BO-264 was added 72 hr after seeding. Organoids were grown in the presence of drug or vehicle for 7 days, and the organoid viability was measured using 3D Cell Titer Glo (Promega).

**Transient transfection with siRNAs and overexpression vectors**

siRNA transfections were done in P/S-free growth medium with reduced serum at a concentration of 100 nM using Lipofectamine 2000^TM^ (Invitrogen) as previously described ^2^. The list of siRNAs used (Dharmacon) are provided in **Supplementary Table S1**. The PLK4 vector was given 24 hr before treatment with BO-264. HEK293T cells were transfected with full length or truncated TACC3 vectors for 24 hr. MCF-7 cells were transfected with full length TACC3 and KIFC1 vectors, 24 hr before induction of CA with cytochalasin D. SK-BR-3 cells were transfected with HDAC2 and MBD2 vectors, along with the full length TACC3 vector for 24 hr.

**APEX2 proximity ligation assay**

JIMT-1 cells were transfected with APEX2-TACC3 vector and synchronized for mitosis and interphase as described above. Biotinylation was performed as previously described^3^. Briefly, cells were treated with 2.5 mM biotin phenol (Iris Biotech) for 1 hr, and 1 mM H_2_O_2_ was added at room temperature (RT) for 2 min. After washing, cells were lysed with RIPA buffer + quenchers (5mM Trolox, 10 mM NaN_3_, and 10mM Sodium Ascorbate). Lysates were sonicated and clarified by centrifugation. Pre-washed Dynabead M-280 Streptavidin beads were incubated with the cell lysate at 4^o^C for 4 hr. After the incubation, beads were washed and boiled in elution buffer, and samples were loaded onto polyacrylamide gel for immunoblotting the interactors.

**Colony formation assay**

Single-cell suspensions of sgCtrl and sgTACC3-expressing JIMT-1 and MDA-MB-231 cells (3x10^3^ cells/well) were plated in a 12-well plate. After 1 week of seeding, cells were fixed with 4% paraformaldehyde for 30 min and stained with 1% crystal violet (Merck) for 20 min at RT. The plates were air dried, and the dye was dissolved in 10% methanol and 10% acetic acid mixture followed by measuring absorbance at 590 nM. Quantification of percentage colonies was done by subtracting background reading of the reagent from the all the readings, followed by normalization to sgCtrl expressing cells. For MCF12A experiment, cells were first transfected with the TACC3 vectors in 6-well format, and then seeded onto 12-well plate, 24 hr after transfection.

**Quantitative RT-PCR analysis**

Total RNA isolation, cDNA synthesis and quantitative real-time PCR assay were performed as previously described ^1,4^. The sequences of the qRT-PCR primers are provided in **Supplementary Table S2**. For data analysis, ΔΔ*C*_T_ method was utilized using Excel (Microsoft).

**Chromatin Immunoprecipitation**

MCF-7 p53-wt and p53^-/-^ cells that were grown to 70% confluency were crosslinked with 1% formaldehyde for 10 min followed by quenching with 125 mM glycine for 5 min. Cells were lysed in 500 µL lysis buffer, and nuclear lysates were extracted. Following the sonication to shear DNA, the chromatin was incubated with the beads together with the FOXM1 antibody (Cell Signaling, 20459) at + 4 °C, overnight with slow agitation. Beads with no antibody was used as the negative control. Samples were washed with low and high salt wash buffers, and Proteinase K treatment was done for 2 h at 62 C with shaking. Samples were incubated at 95 °C for 10 min and separated from the beads using a magnetic separator. DNA was isolated, and RT-PCR was performed using different primers targeting the predicted FOXM1 binding sites on the TACC3 and KIFC1 promoters (**Supplementary Table S3**). The results were normalized to bead only control and represented as fold enrichment.

**Western blotting**

Protein isolation and Western blotting were done as previously^1,5^ described. Briefly, proteins were extracted using RIPA lysis buffer with the addition of protease and phosphatase inhibitor cocktails. For protein isolation from tumors, the lysates were sonicated on ice for 10 seconds to disrupt the tissue. Equal amounts of protein were separated using 10% SDS-PAGE gel. Separated proteins were transferred to PVDF membranes (Bio-Rad) using a Trans-Blot turbo transfer system (Bio-Rad) and incubated with primary antibodies (**Supplementary Table S4**) overnight at 4^o^C followed by secondary antibody incubation, and signal detection by chemiluminescence. Images were acquired using Image Lab Software (Bio-Rad).

**Immunoprecipitation**

After synchronizing JIMT-1 cells in mitosis or interphase, cells were treated with 5 µM of BO-264 for 4 hr. Cells were lyzed in lysis buffer (50 mM TrisHCl pH=7.0, 150 mM NaCl, 0.2% NP40, 7.5% glycerol, protease and phosphatase inhibitor cocktail), and clarified by centrifugation. 1 mg protein for each condition was incubated with antibody-coated Dynabeads Protein G (Invitrogen). Antibody dilutions are provided in **Supplementary Table S4**. Beads were washed and resuspended in 60 µL 1X SDS sample loading buffer, boiled for 10 min at 70 ^o^C and loaded onto polyacrylamide gel.

**Drug affinity responsive target stability**

Drug affinity responsive target stability (DARTS) was performed as described previously^4^. Briefly, HEK-293T cells were transfected with full length or truncated TACC3 vectors for 48 hr cells and lysed in mild RIPA buffer with 1% NP40. Cell lysate–drug mixtures were incubated at room temperature on a shaker for 60 minutes to allow binding, and each sample was mixed with 2 μL of 8 ng/μL pronase solution (Sigma Aldrich) or buffer only (undigested) and incubated at room temperature for 15 min. Protein digestion was stopped by adding protease inhibitor cocktail (Roche), and lysates were mixed with 8 μL of 4× SDS loading buffer and heated at 70°C for 10 minutes. SDS-PAGE was performed with the N-terminal-specific anti-TACC3 antibody (SantaCruz, sc-376883) for detecting full length and N-terminal regions, while C-terminal-specific anti-TACC3 antibody (Invitrogen, PA5-36349) was used for detecting C-terminal region.

**Chromatin fractionation**

JIMT-1 cells were treated with 5 µM BO-264 for 4 hr, then lyzed in the lysis buffer containing 150 mM KCl, 50 mM HEPES pH=7.4, 2.5 mM MgCl_2_, 5 mM EDTA pH=8, 3 mM DTT, 0.5% Triton X-100, 10% glycerol and protease inhibitor for 45 min on ice. Centrifugation was done at 16,000 g for 15 min to pellet the chromatin. Supernatant was collected as the cytosolic fraction, while the insoluble chromatin was washed once with lysis buffer, resuspended in lysis buffer and then sonicated. Protein concentration was measured using BCA Protein Assay (Thermo Scientific), and equal amounts of protein from cytosol and chromatin factions were mixed with SDS loading dye and boiled at 95 ^0^C for 10 min.

**Immunofluorescence and quantification**

Immunofluorescence staining of fixed cultured cells was performed as previously described^1,4^. Dilutions for the primary and secondary antibody incubations are provided in **Supplementary Table S4**. Images en with Zeiss LSM700 Confocal Microscopy and analyzed using ImageJ software ^6^. Quantification of cells with multipolar or bipolar spindles was done by counting the number of mitotic cells with >2 scattered centrosomes (multipolar) or with 2 or more centrosomes clustered in a bipolar manner (bipolar) as determined by staining centrin 2 or γ-tubulin as centrosomal markers. Then, the ratio to total number of mitotic cells was calculated and represented as a percentage. At least 100 cells for treatment groups and 20-50 cells for the control groups were quantified. Quantification of the colocalization between TACC3 and MBD2/HDAC2 was done by calculating the closeness factor as defined by the log transformed value of 1/[incremental change in the ratio of the intensities of TACC3 and HDAC2/MBD2]. At least 200 cells were quantified for the colocalization analysis.

Immunofluorescence staining of FFPE TMA slides and MDA-MB-231 xenograft tumor slides were performed by deparaffinization at 60 ℃ for 1 hr, followed by rehydration in citrisolv for 5 min (3 times), 100% ethanol for 5 min (twice), 95% ethanol for 5 min (twice), deionized water for 5 min (twice). Antigen retrieval was done with Tris-EDTA pH=9 at 96 ℃ for 15 min, followed by cooling down to room temperature for 30 min, and washing with TBST for 5 min. Blocking was done at room temperature with Buffer W (IBA-Lifesciences) for 30 min. TACC3 and pan-cytokeratin immunofluorescence was employed using the OPAL multiplexing method based on Tyramide Signal Amplification (TSA) with Opal 620 and Opal 690. For α-tubulin/γ-tubulin dual staining in MDA-MB-231 xenografts or γ-tubulin staining in TMAs, consecutive slides were incubated with the primary antibodies at a dilution of 1:500 for overnight, at +4 ℃ (**Supplementary Table S4**), followed by 3 times washing in PBS-Tween for 5 min and incubation with the Alexa fluor-647 and Alexa fluor-488 with conjugated secondary antibodies at a dilution of 1:100 for 2.5 hr at 37 ℃. DAPI was used for nuclear counterstaining. The entire core areas were scanned using the Vectra® Polaris™ Automated Quantitative Pathology Imaging System (Akoya Biosciences) at 20X for TACC3/Cytokeratin and at 40X for γ-tubulin. Segmentation of tissue was done using Cytokeratin as the epithelial marker. Cells were then phenotyped based on TACC3 and cytokeratin positivity using inForm® Tissue Analysis Software (v[2.6.0], Akoya Biosciences). To quantify CA, the autofluoresence-corrected images were uploaded to QuPath software (v[0.4.2])^7^. Epithelial tumor cells were classified based on DAPI which could detect cytokeratin-positive cells with more than 95% accuracy. CA quantification was done by taking the sum of the number of cells with more than 2 γ-tubulin foci and with centrosomes larger than 1.44 um^2^ which is the average size of centrosomes in normal breast, as previously described^8,9^.

**Annexin V/PI staining and cell cycle assay**

Annexin V/PI staining was done as previously described^1,4^. Briefly, counted cells for each condition were washed with PBS and incubated with 1.5 µL of FITC-conjugated Annexin V and PI for 30 min. Data collection was done with BD FACSDiva software (BD, NJ, USA), and analysis with De Novo FCS Express software. For cell cycle analysis, cells were fixed and permeabilized in 70% ethanol and then incubated with p-Histone H3 antibody and anti-rabbit Alexa-Fluor 488 antibody for 45 min at RT, sequentially. Lastly, cells were incubated in DAPI for 15 min at RT followed by data collection and analysis.

**
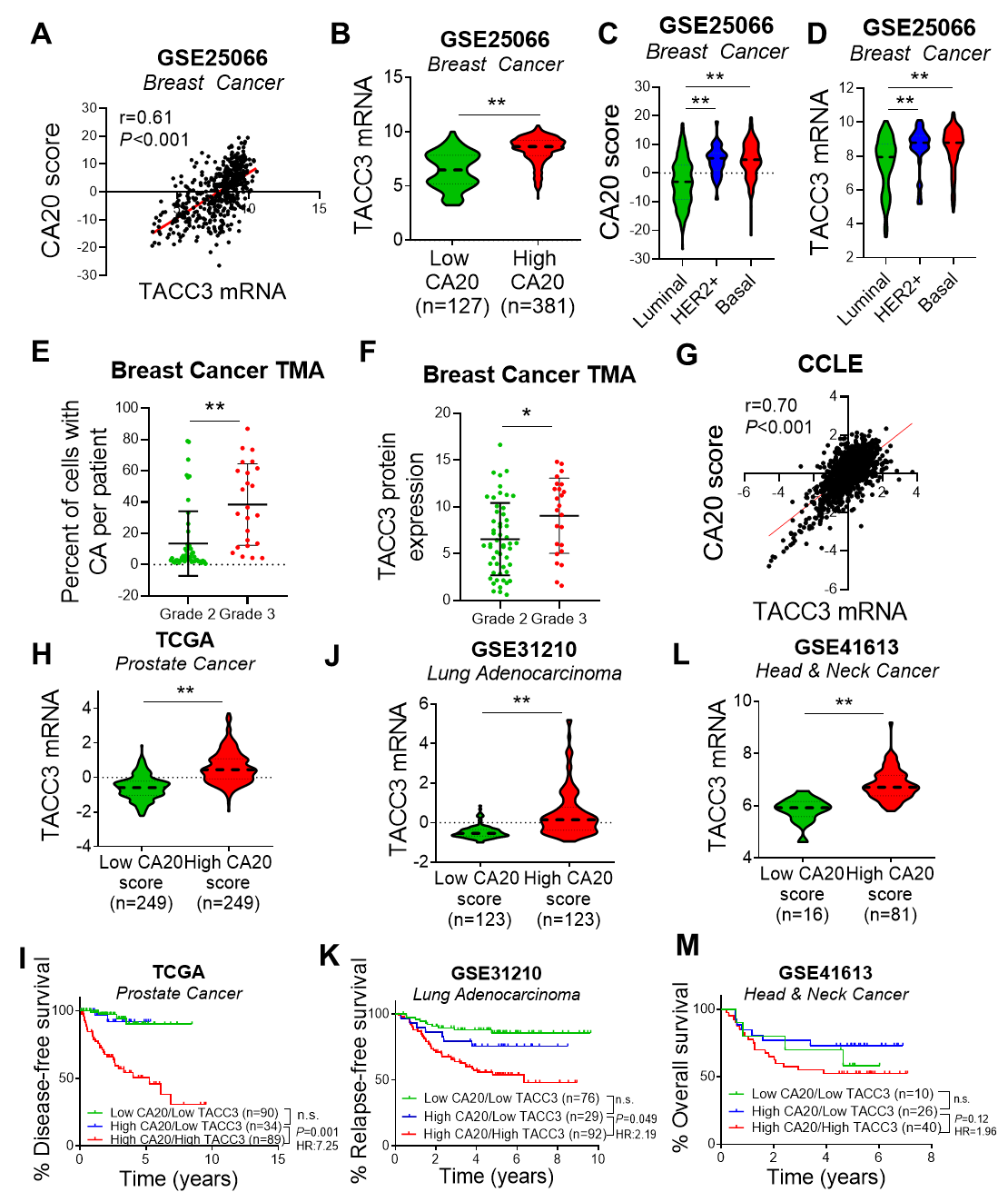
Supplementary Figures**

**Supplementary Fig. S1. TACC3 is upregulated in highly aggressive tumors with CA and associated with worse clinical outcome. A.** Correlation of TACC3 mRNA expression with CA20 score in breast cancer tumors in GSE25066 dataset. **B.** TACC3 mRNA expression in low vs. high CA tumors in GSE25066 dataset. **C, D.** Expression of CA20 score (**C**) and TACC3 mRNA (**D**) in different breast cancer subtypes in GSE25066 dataset. **E****.** Percent of cells with CA per patient in grade 2 vs. grade 3 breast cancer patients from tissue microarray (BR1902, TissueArray). **F.** TACC3 protein expression in grade 2 vs. grade 3 breast cancer patients from tissue microarray (BR1902, TissueArray). **G.** Correlation of TACC3 mRNA expression with CA20 score in cancer cell lines in CCLE dataset. **H,** **I.** TACC3 mRNA expression (I) and disease-free survival (J) in low vs. high CA tumors of prostate. **J, K.** TACC3 mRNA expression (K) and relapse-free survival (L) in low vs. high CA tumors of lung. **L, M.** TACC3 mRNA expression (M) and overall survival (N) in low vs. high CA tumors of head & neck. CA, centrosome amplification. *, *P*<0.05; **. *P*<0.01.

**
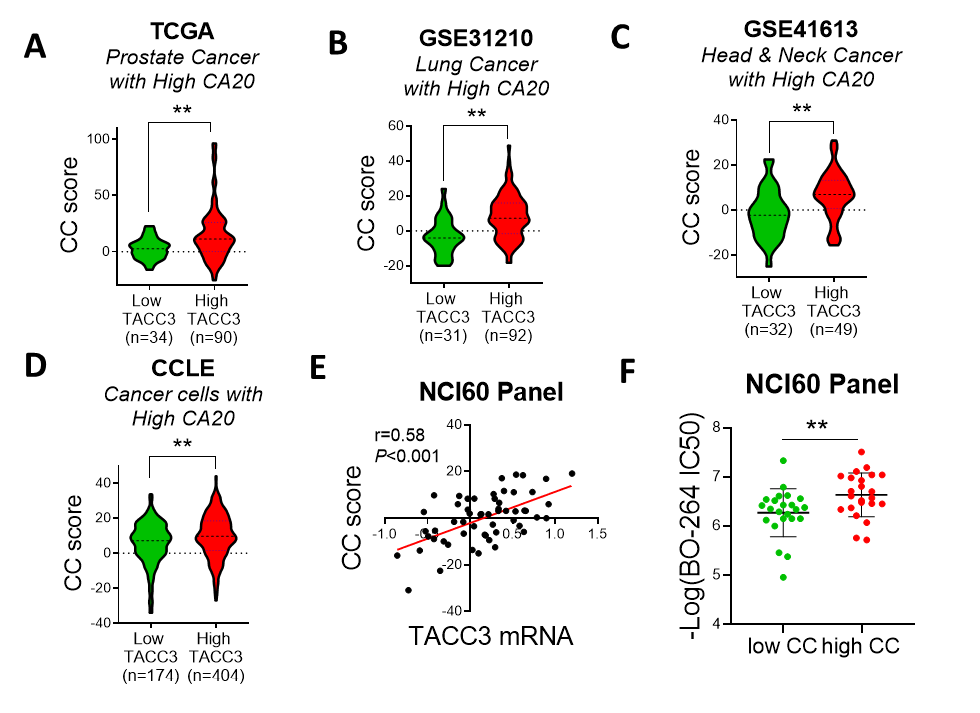
**

**Supplementary Fig. S2. TACC3 expression correlates with CC in tumors and cancer cell lines, and high CC correlates with higher sensitivity to TACC3 inhibition.** **A-C.** Expression of CC score in prostate (**A**), lung (**B**), and head & neck (**C**) tumors bearing high CA20 expression and separated based on low vs. high TACC3 expression. **D.** Expression of CC score in cancer cell lines with high CA20 expression in CCLE dataset separated based on low vs. high TACC3 expression. **E.** Correlation of TACC3 expression with CC score in NCI60 cell line panel. **F.** BO-264 IC50 values (-log) in NCI60 cells separated based on CC score. **. *P*<0.01.

**
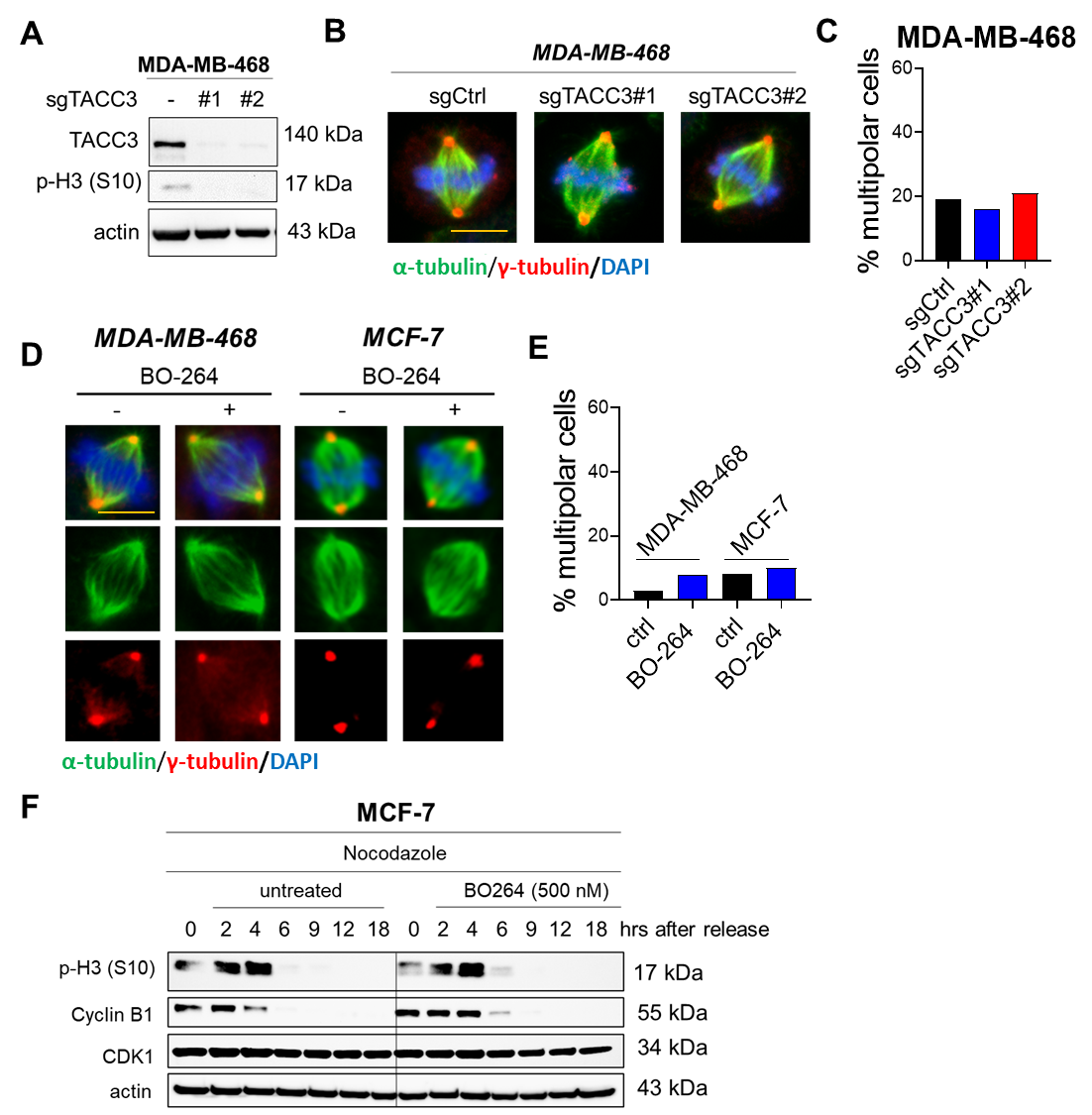
**

**Supplementary Fig. S3. TACC3 inhibition does not lead to spindle defects and mitotic arrest in cancer cells without CA. A** Western blot analysis of TACC3 and the mitotic arrest markers, p-H3 (S10) in MDA-MB-468.sgCtrl vs. sgTACC3 cells. **B** Analyses of spindle formation in MDA-MB-468.sgCtrl vs. sgTACC3 cells as shown by α- (spindle, green) and γ- (centrosome, red) tubulin staining. Scale bar = 10 µm. **C** Quantification of mitotic cells with multipolar spindles from B. **D** Analyses of spindle formation in BO-264-treated MDA-MB-468 and MCF-7 cells as shown by α- (spindle, green) and γ- (centrosome, red) tubulin staining. Scale bar = 10 µm. **E** Quantification of mitotic cells with multipolar spindles from D. **F** Western blot analysis of mitosis markers in MCF-7 cells synchronized at mitosis using nocodazole followed by release into fresh vs. BO-264-containing media. Actin is used as a loading control.

**
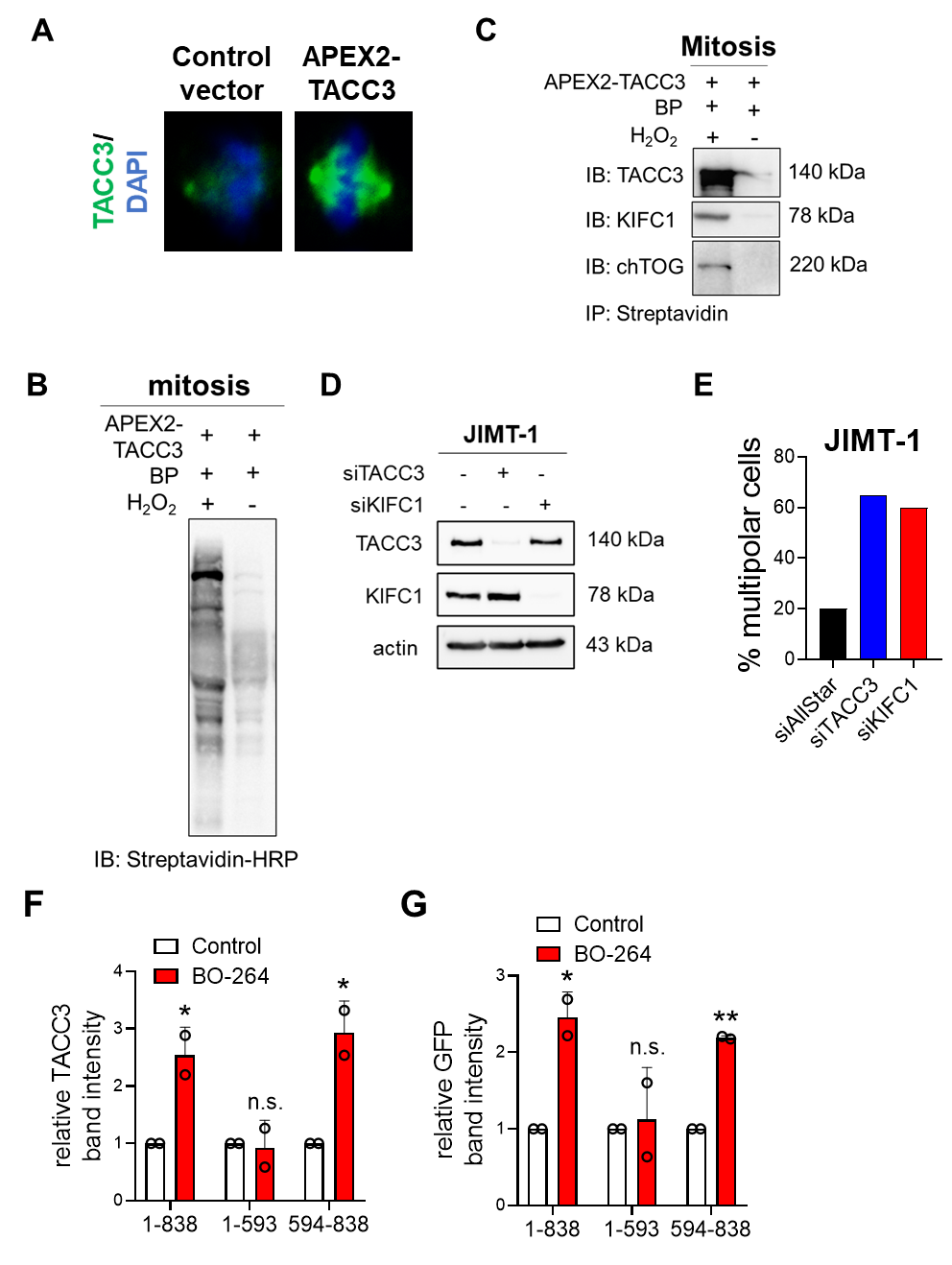
**

**Supplementary Fig. S4. TACC3 interacts with KIFC1 on its C-terminal TACC domain and siRNA-mediated inhibition of TACC3 or KIFC1 leads to centrosome de-clustering and multipolar cells.** **A.** Immunofluorescence staining of APEX2-TACC3 in mitotic JIMT-1 cells. **B.** Western blot analysis of biotinylated proteins upon H_2_O_2_ in mitotic JIMT-1 cells overexpressing APEX2-TACC3. **C** Western blot analysis of KIFC1 and the known interactor, chTOG upon biotinylation by H_2_O_2_ in mitotic JIMT-1 cells followed by Streptavidin pulldown. **D** Western blot analysis of TACC3 and KIFC1 to validate the siRNA-mediated knockdowns. **E** Quantification of mitotic cells with multipolar mitosis upon knockdown of TACC3 or KIFC1. **F, G** normalized band intensities of the TACC3 (F) and GFP (G) in the DARTS assay showing differential binding of GFP-tagged truncated TACC3 vectors to the TACC3 inhibitor, BO-264. The graphs are drawn by taking the average of two biological replicates. *. *P*<0.05; **, *P*<0.01.

**
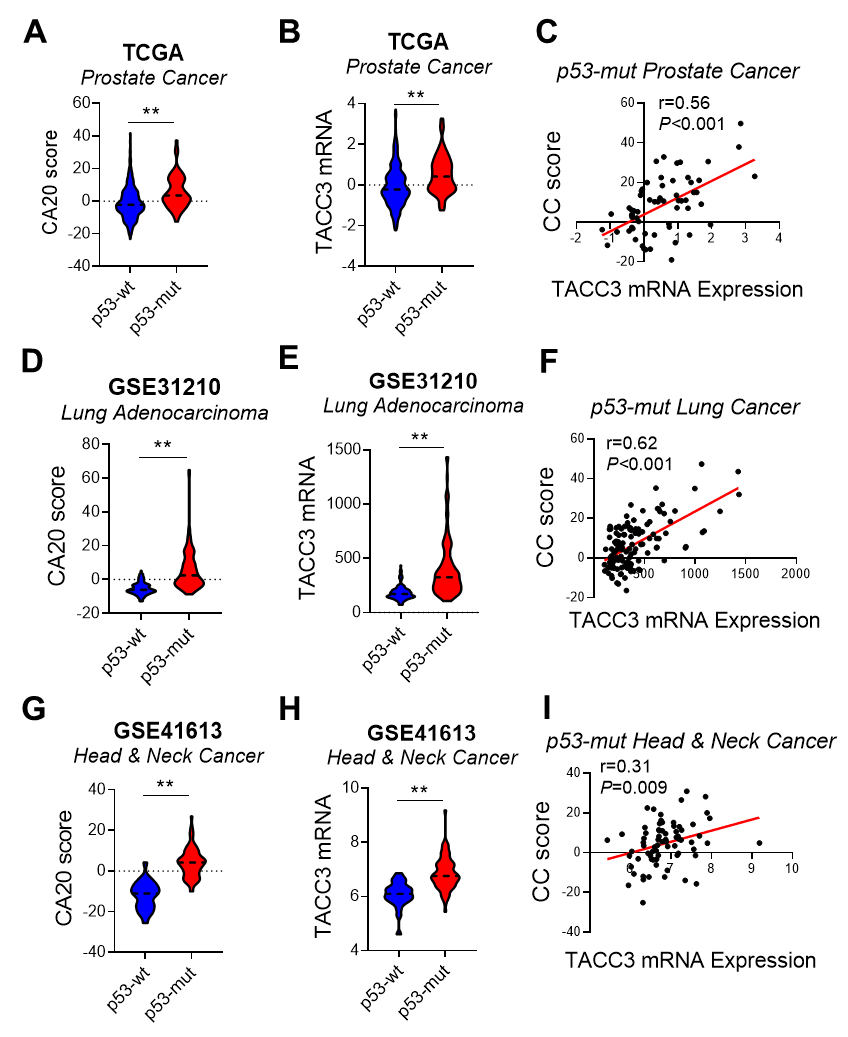
**

**Supplementary Fig. S5. Correlation of TACC3 with CC in p53-mut cancer patients. A-C.** CA20 score (A) and TACC3 (B) expression in p53-wt vs. p53-mut prostate cancer patients in TCGA dataset, and the correlation between TACC3 expression and CC score in p53-mut prostate cancer patients (C). **D-F.** CA20 score (D) and TACC3 (E) expression in p53-wt vs. p53-mut lung cancer patients in GSE31210 dataset, and the correlation between TACC3 expression and CC score in p53-mut lung cancer patients (F). **G-I.** CA20 score (G) and TACC3 (H) expression in p53-wt vs p53-mut head & neck cancer patients in GSE41613 dataset, and the correlation between TACC3 expression and CC score in p53-mut head & neck cancer patients (I). **. *P*<0.01.

**
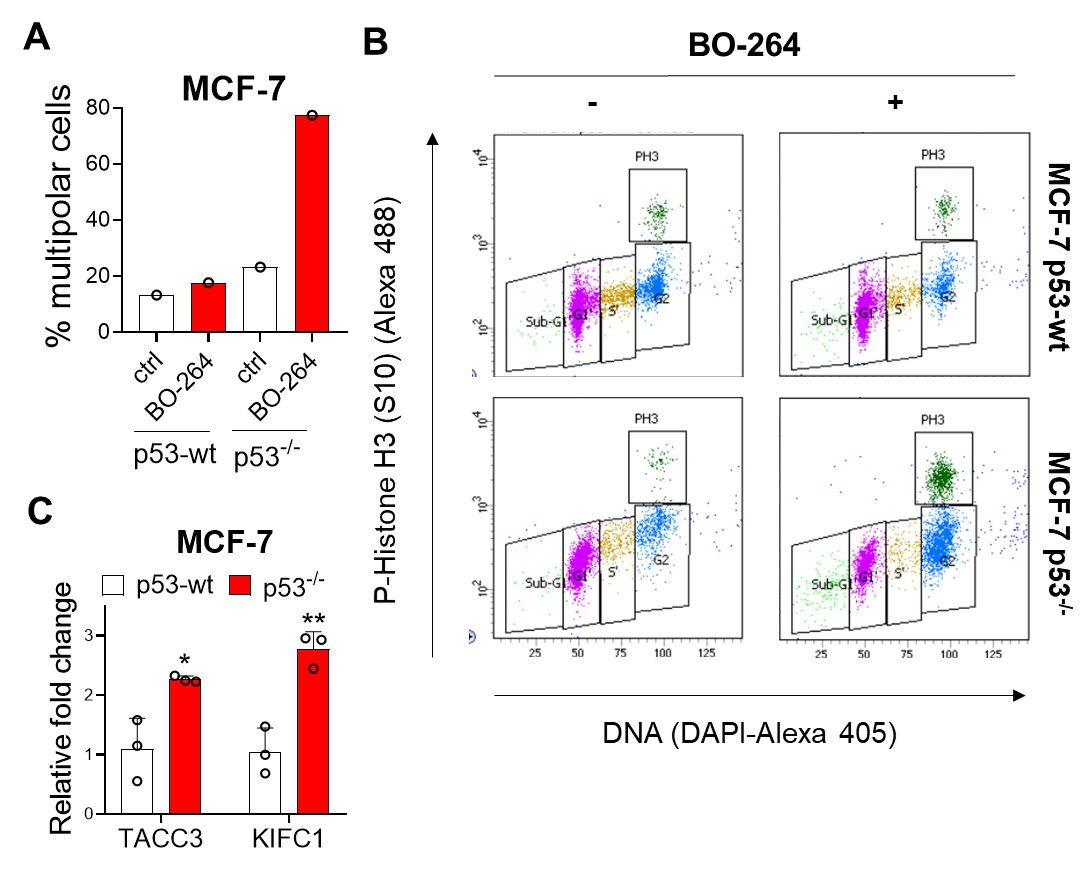
**

**Supplementary Fig. S6. Effects of TACC3 inhibition on multipolar mitosis and mitotic arrest and the mRNA expressions of TACC3 and KIFC1 in MCF-7 p53-wt vs. p53^-/-^ cells. A.** Quantification of mitotic cells with multipolar spindles in BO-264-treated MCF-7 p53-wt vs p53^-/-^ cells. **B.** Flow cytometry analysis of DAPI and p-Histone H3 in MCF-7 p53-wt vs. p53^-/-^ cells treated with 2 µM BO-264 for 24 hrs. Population of cells at different cell cycle phases are depicted with different colors and shown in boxes. **C.** qRT-PCR analysis of TACC3 and KIFC1 in MCF-7 p53-wt vs p53^-/-^ cells. *. *P*<0.01. **. *P*<0.01.

**
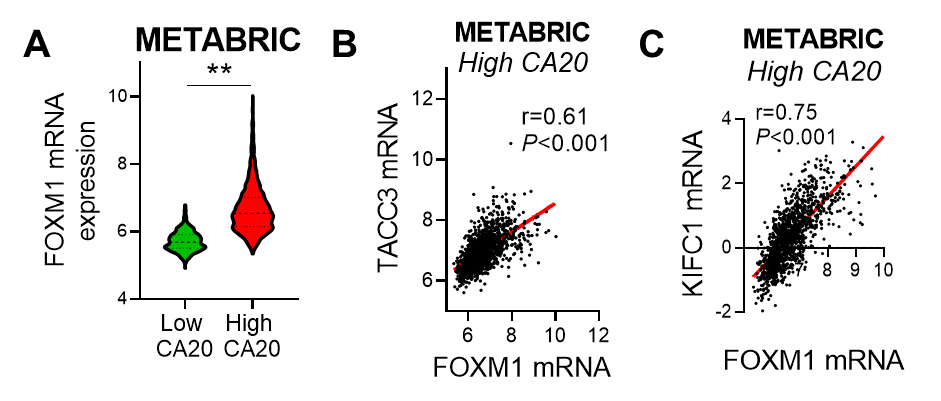
**

**Supplementary Fig. S7.** **The association of CA, TACC3 and KIFC1 with FOXM1 in breast cancer patients.** **A.** FOXM1 mRNA expression in low vs. high CA20 expressing breast tumors from METABRIC. **B, C.** Correlation of FOXM1 expression with TACC3 (B) and KIFC1 (C) in breast cancer patients with high CA20 expressing from METABRIC. **. *P*<0.01.


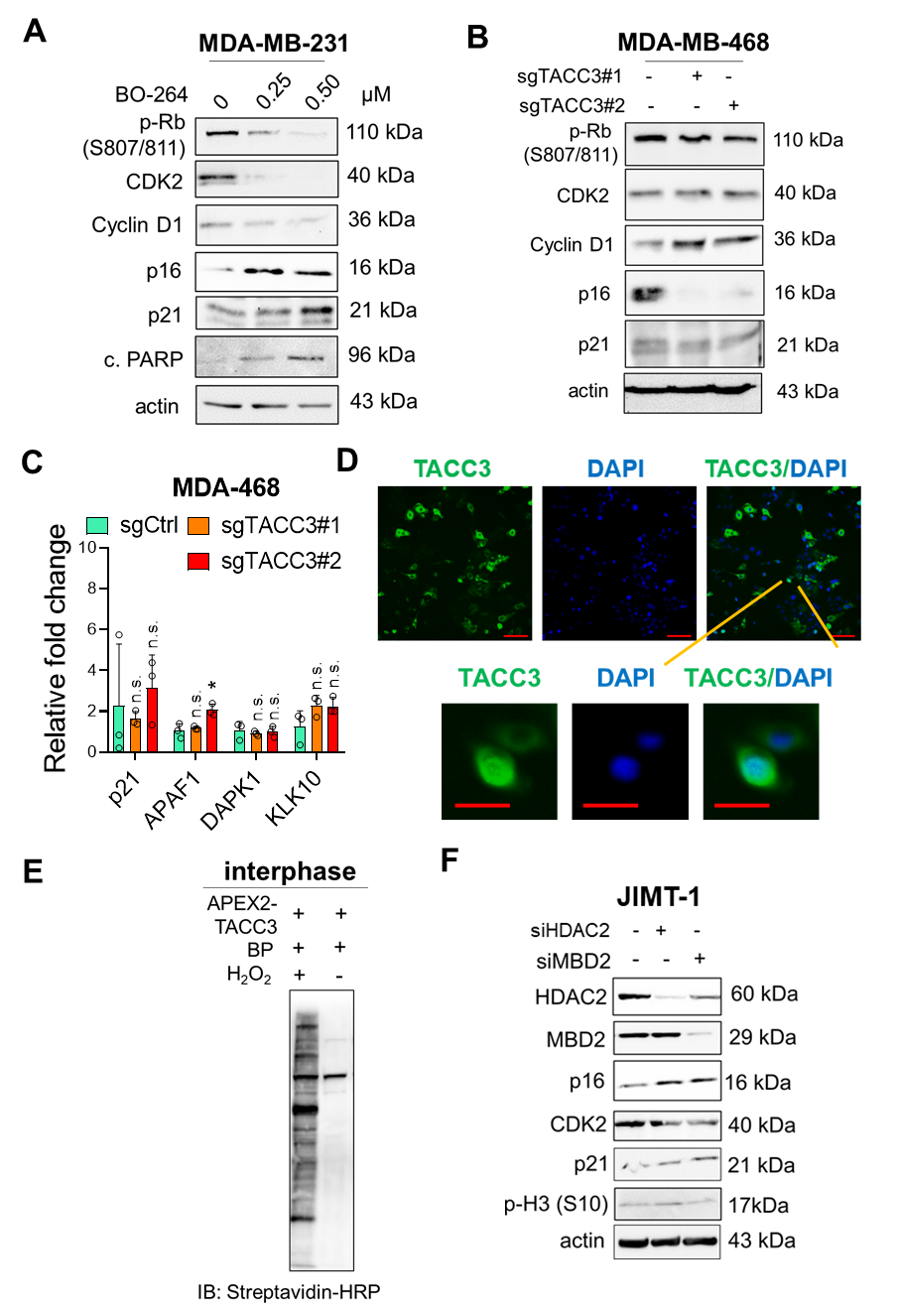


**Supplementary Fig. S8. Validating the interaction of TACC3 with the NuRD complex and CA-specific G1 arrest upon inhibiting TACC3 or NuRD complex members. A, B.** Western blot analysis of G1/S progression markers and CDK inhibitors in MDA-MB-231 (A) and MDA-MB-468.sgCtrl vs. sgTACC3 cells (B) upon TACC3 inhibitor treatment or TACC3 knockout, respectively. **C.** qRT-PCR of NuRD complex targets in MDA-MB-468.sgCtrl vs. sgTACC3 cells. **D.** Western blot analysis of biotinylated proteins upon H_2_O_2_ in interphase JIMT-1 cells overexpressing APEX2-TACC3. **E.** Immunofluorescence staining of TACC3 in interphase JIMT-1 cells overexpressing APEX2-TACC3. DAPI is a marker for nucleus. Scale bar = 10 µm. **F**. Western blot analysis of G1/S progression markers, CDK inhibitors and mitotic arrest marker, p-H3 in JIMT-1 cells transfected with siHDAC2 and siMBD2. *. *P*<0.05; n.s., not significant.


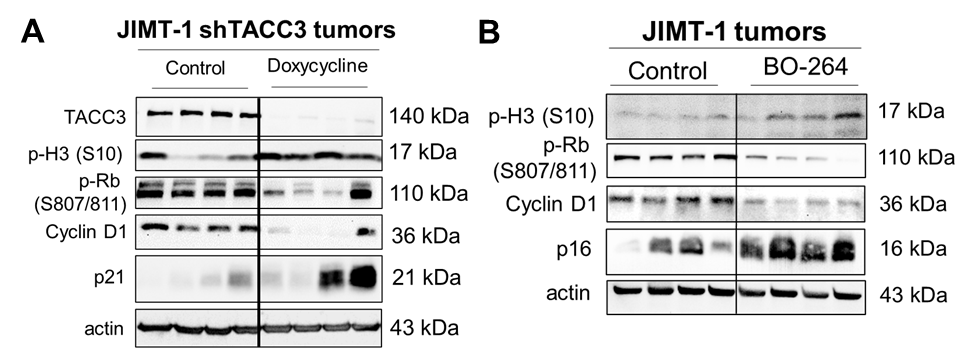


**Supplementary Fig. S9. Changes of mitotic and G/S progression markers upon knockdown or pharmacologic inhibition of TACC3 in JIMT-1 xenografts with CA.** **A, B.** Western blot analysis of TACC3, mitotic progression and G1/S progression markers and CDK inhibitors in doxycycline-induced (1 mg/kg for 5 days) shTACC3 tumors (A) or BO-264-treated (50 mg/kg, once) (B) JIMT-1 (CA) xenograft tumors. n=4.


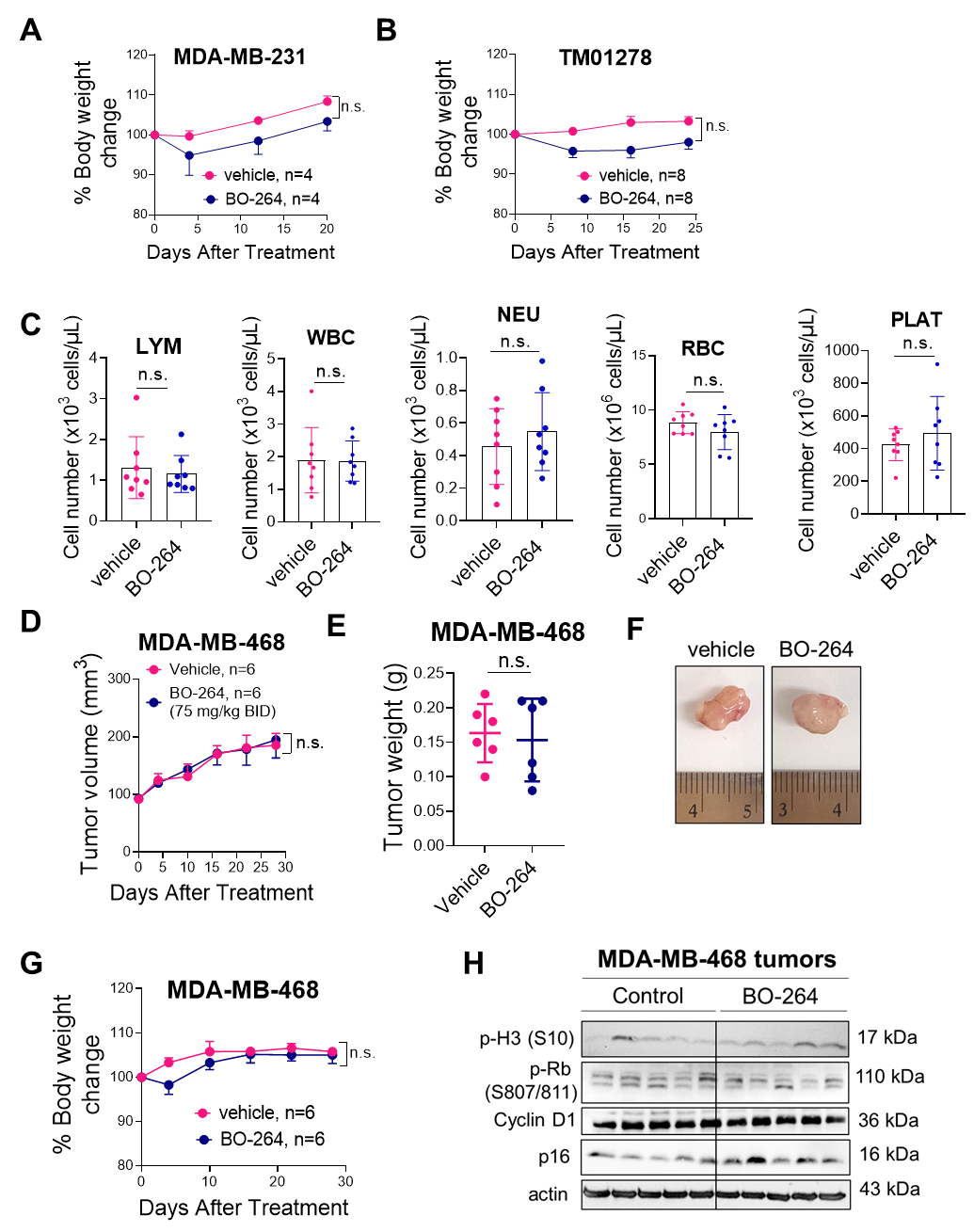


**Supplementary Fig. S10. Tumor growth, percent body weight change and blood cell count in BO-264-treated MDA-MB-231 and MDA-MB-468 xenografts and TM01278. A-C.** Percent body weight change of MDA-MB-231 xenografts (**A**) and TM01278 PDXs (**B**) upon treatment with BO-264 twice daily at a dose of 75 mg/kg (p.o.). **C.** Blood cell count of the BO-264-treated TM01278 mice at the end of the experiment. **D-F.** Tumor growth (D), tumor weight (E) and representative images of tumors (F) at the end of the experiment from BO-264-treated (75 mg/kg, bidaily, p.o.) MDA-MB-468 xenografts. **G.** Percent body weight change of MDA-MB-468 xenografts upon treatment with BO-264 twice daily at a dose of 75 mg/kg (p.o.). **H.** Western blot analysis of mitotic progression and G1/S progression markers and CDK inhibitors in BO-264-treated (50 mg/kg, once) MDA-MB-468 (non-CA) xenograft tumors. n=5. n.s., not significant.

**Supplementary Tables**

**Supplementary Table S1.** Sequences of siRNAs used.

| **Gene Name** | **NCBI Gene ID** | **Company** | **Catalog number** | **sequence** |
| --- | --- | --- | --- | --- |
| TACC3 | 10460 | Dharmacon | D-004155-02-0002 | GAGCGGACCUGUAAAACUA |
| KIFC1 | 3833 | Dharmacon | D-004958-02-0002 | GUGCUAAGAUGCUCAUGUU |
| HDAC2 | 3066 | Dharmacon | D-003495-05-0002 | CGGUAUCAUUCCAUAAAUA |
| MBD2 | 8932 | Dharmacon | D-011555-20-0002 | GGGCUAAGUGCUGGCAAGA |
| FOXM1 | 2305 | Dharmacon | D-009762-02-0002 | GGACCACUUUCCCUACUUU |

**Supplementary Table S2.** Sequences of qRT-PCR primers.

| **Gene Name** | **NCBI Gene ID** |  | **Primer sequence** |
| --- | --- | --- | --- |
| ACTB | 60 | Forward | 5’-CCAACCGCGAGAAGATGA-3’ |
|  |  | Reverse | 5’-CCAGAGGCGTACAGGGATAG-3’ |
| HPRT | 3251 | Forward | 5’-TGACCTTGATTTATTTTGCATACC-3’ |
|  |  | Reverse | 5’-CGAGCAAGACGTTCAGTCCT-3’ |
| DAPK1 | 1612 | Forward | 5’-TGTCTTCCACCAACTCCAGCAG-3’ |
|  |  | Reverse | 5’-AAATCGCCAACTCCATTCAAATAAGC-3’ |
| KLK10 | 5655 | Forward | 5’-GCCCGGAGAGTGAAGTACAA-3’ |
|  |  | Reverse | 5’-GTAAACACCCCACGAGAGGA-3’ |
| APAF1 | 317 | Forward | 5’-CACGTTCAAAGGTGGCTGAT-3’ |
|  |  | Reverse | 5’-TGGTCAACTGCAAGGACCAT-3’ |
| CDKN1A | 1026 | Forward | 5’-TGAGCCGCGACTGTGATG-3’ |
|  |  | Reverse | 5’-GTCTCGGTGACAAAGTCGAAGTT-3’ |
| CDKN2A | 1029 | Forward | 5’-GAGCAGCATGGAGCCTTC-3’ |
|  |  | Reverse | 5’-CCTCCGACCGTAACTATTCG-3’ |
| TACC3 | 10460 | Forward | 5’-GTCTGTCTGTCCTGTCTGATTC-‘3 |
|  |  | Reverse | 5’-GACAGTGGAGCAGAAGACTAAA-‘3 |
| KIFC1 | 3833 | Forward | 5’-TGAGCAACAAGGAGTCCCAC-3’ |
|  |  | Reverse | 5’-TCACTTCCTGTTGGCCTGAG-3’ |

**Supplementary Table S3.** Sequences of ChIP qRT-PCR primers.

| **PrimerName** | **Gene Target** |  | **Primer sequence** |
| --- | --- | --- | --- |
| TACC3-1 | TACC3 | Forward | 5’-CTGGCCAAAGCTATGAAGGTA-3’ |
|  |  | Reverse | 5’-TGAGGAGGAGAGCACCTATT-3’ |
| TACC3-2 | TACC3 | Forward | 5’-AGAATGAGTCTGCAGGTCTTAAA-3’ |
|  |  | Reverse | 5’-GATCTTCCGGTAACTTCTGGTG-3’ |
| TACC3-3 | TACC3 | Forward | 5’-CTCTGATGTGGAAGTTCCTTGG-3’ |
|  |  | Reverse | 5’-AGGCTGAGAGGGTGAAGG-3’ |
| TACC3-4 | TACC3 | Forward | 5’-CTCACGTCACACGCAGAG-3’ |
|  |  | Reverse | 5’-TCAAGCAGAAGTCAAGAGACG-3’ |
| KIFC1-1 | KIFC1 | Forward | 5’-ACGATTGGTCCCTGCGT-3’ |
|  |  | Reverse | 5’-GCAGGGTAGAGAACTCGCA-3’ |
| KIFC1-2 | KIFC1 | Forward | 5’-TCGGCCTTTGTCATGTCTAC-3’ |
|  |  | Reverse | 5’-CTCCAGAAACTTGAAGCTAGGG-3’ |

**Supplementary Table S4.** List of antibodies used in Western blot (WB), immunofluorescence (IF), immunoprecipitation (IP) and IF/IHC experiments.

| **Antibody** | **Provider** | **Catalog number** | **WB dilution** | **IF dilution** | **IP/ChIP dilution** | **Histology IHC/IF** |
| --- | --- | --- | --- | --- | --- | --- |
| Alexa Fluor® 488 anti-mouse | Life Technologies | A-11001 | - | 1:200 | - | - |
| Alexa Fluor® 647 anti-rabbit | Life Technologies | A-31573 | - | 1:200 | - | 1:1000 (IF) |
| Beta-actin | MP Biomedicals | 691001 | 1:10000 | - | - | - |
| Cleaved PARP | Cell Signaling Technology | 5625 | 1:1000 | - | - | - |
| Alpha-tubulin | Santa Cruz | 32293 | 1:1000 | 1:500 | - | 1:500 (IF) |
| Gamma-tubulin | Sigma Aldrich | T3195 | - | 1:200 | - | 1:500 (IF) |
| p-Histone H3 (Ser10) | Cell Signaling Technology | 4056 | 1:1000 | - | - | - |
| Histone H3 | Cell Signaling Technology | 4499 | 1:1000 | - | - | - |
| APAF1 | Cell Signaling Technology | 8969 | 1:1000 | - | - | - |
| CDK2 | Cell Signaling Technology | 18048 | 1:1000 | - | - | - |
| Cyclin E1 | Cell Signaling Technology | 4129 | 1:1000 | - | - | - |
| Cyclin D1 | Cell Signaling Technology | 2922 | 1:1000 | - | - | - |
| Cyclin B1 | Cell Signaling Technology | 4138 | 1:1000 | - | - | - |
| p16 | Cell Signaling Technology | 80772 | 1:1000 | - | - | - |
| p21 | BD Biosciences | BDB554228 | 1:1000 | - | - | - |
| TACC3 | Santa Cruz | 376883 | 1:1000 | 1:400 | 1:10 (IP) | 1:400 (IF) 1:100 (IHC) |
| TACC3 | Invitrogen | PA5-36349 | 1:1000 | - | - | - |
| FOXM1 | Cell Signaling Technology | 20459 | - | - | 1:100 (ChIP) | - |
| KIFC1 | Abcam | ab172620 | 1:1000 | 1:400 | 1:100 (IP) | - |
| MBD2 | Abcam | ab188474 | 1:1000 | 1:400 | 1:100 (IP) | - |
| HDAC2 | Abcam | ab16032 | 1:1000 | 1:400 | 1:100 (IP) | - |
| Clathrin | Abcam | ab21679 | 1:1000 | - | - | - |
| GFP | Genetex | GTX628528 | 1:1000 | - | - | - |
| Cytokeratin | Roche | 760-2595 | - | - | - | 1:10000 (IF) |
|  |  |  |  |  |  |  |

**Supplementary References**

1 Saatci, O. et al. Targeting lysyl oxidase (LOX) overcomes chemotherapy resistance in triple negative breast cancer. Nature communications. 2020;11:2416.

2 Assidicky, R. et al. Targeting HIF1-alpha/miR-326/ITGA5 axis potentiates chemotherapy response in triple-negative breast cancer. Breast Cancer Res Treat. 2022;

3 Tan, B. et al. An Optimized Protocol for Proximity Biotinylation in Confluent Epithelial Cell Cultures Using the Peroxidase APEX2. STAR Protoc. 2020;1:100074.

4 Akbulut, O. et al. A Highly Potent TACC3 Inhibitor as a Novel Anticancer Drug Candidate. Mol Cancer Ther. 2020;19:1243-1254.

5 Saatci, O. et al. Targeting PLK1 overcomes T-DM1 resistance via CDK1-dependent phosphorylation and inactivation of Bcl-2/xL in HER2-positive breast cancer. Oncogene. 2018;37:2251-2269.

6 Schindelin, J. et al. Fiji: an open-source platform for biological-image analysis. Nat Methods. 2012;9:676-682.

7 Bankhead, P. et al. QuPath: Open source software for digital pathology image analysis. Scientific reports. 2017;7:16878.

8 Mittal, K. et al. Amplified centrosomes and mitotic index display poor concordance between patient tumors and cultured cancer cells. Scientific reports. 2017;7:43984.

9 Patel, N. et al. Integrated genomics and functional validation identifies malignant cell specific dependencies in triple negative breast cancer. Nature communications. 2018;9:1044.
