# Supplementary material for: Targeting TACC3 represents a novel vulnerability in highly aggressive breast cancers with centrosome amplification
Source: Cell Death Differ. 2023 Mar 2;30(5):1305–19. doi: 10.1038/s41418-023-01140-1 (PMC10154422; doi:10.1038/s41418-023-01140-1)

Figure 1I

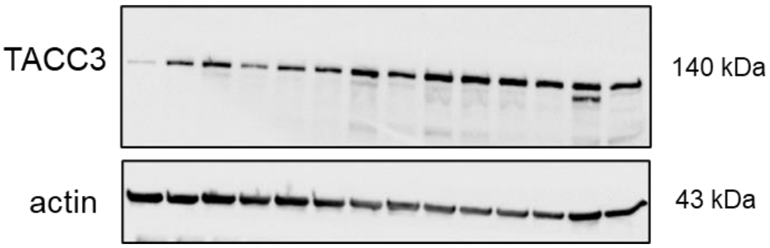

Figure 1L

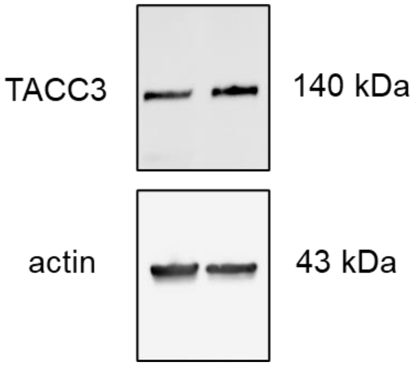

Figure 2C

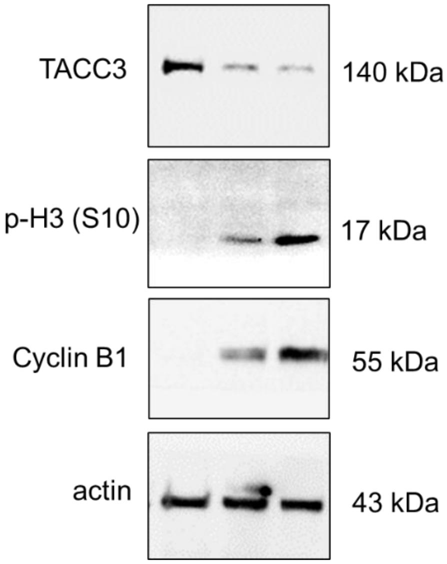

Figure 2H

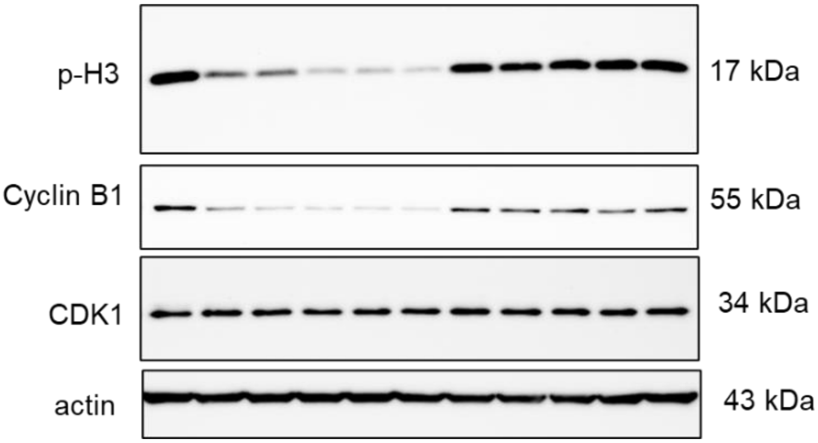

Figure 3H

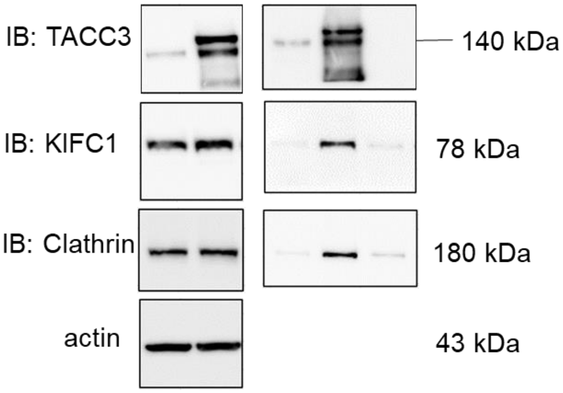

Figure 3I

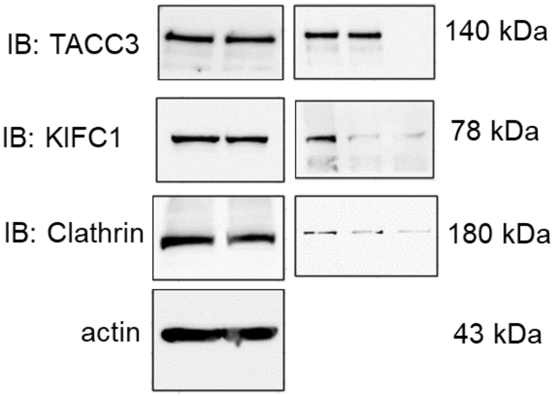

Figure 3O

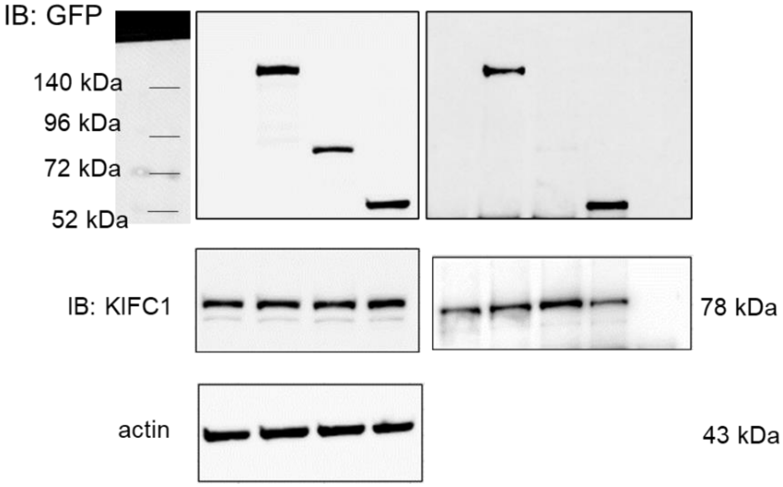

Figure 3P

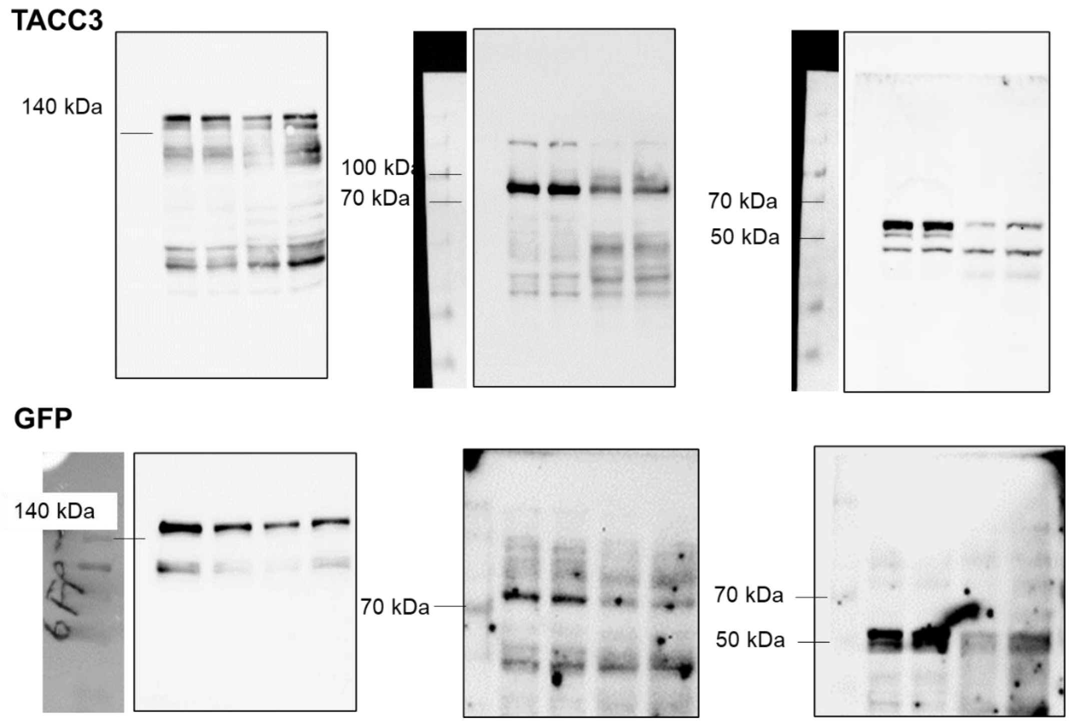
TACC3
140 kDa
100 kDa
70 kDa
70 kDa
50 kDa
GFP
140 kDa
70 kDa
70 kDa
50 kDa

Figure 4E

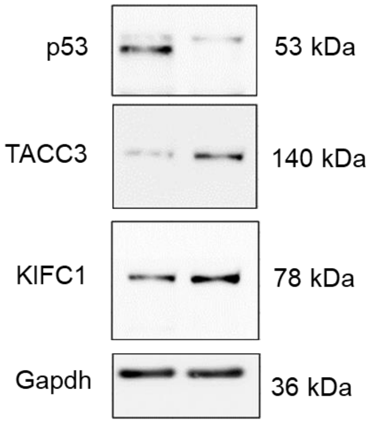

Figure 4H

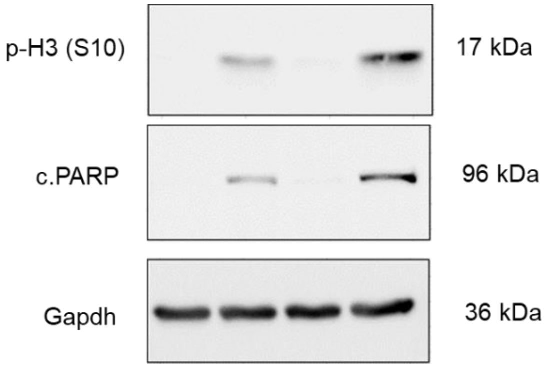

Figure 4N

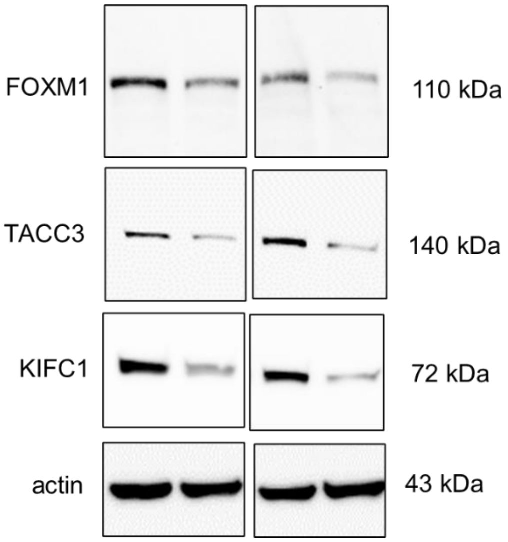

Figure 4O

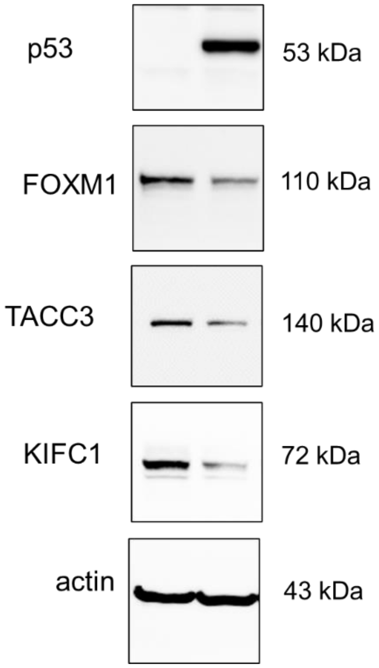

Figure 5A

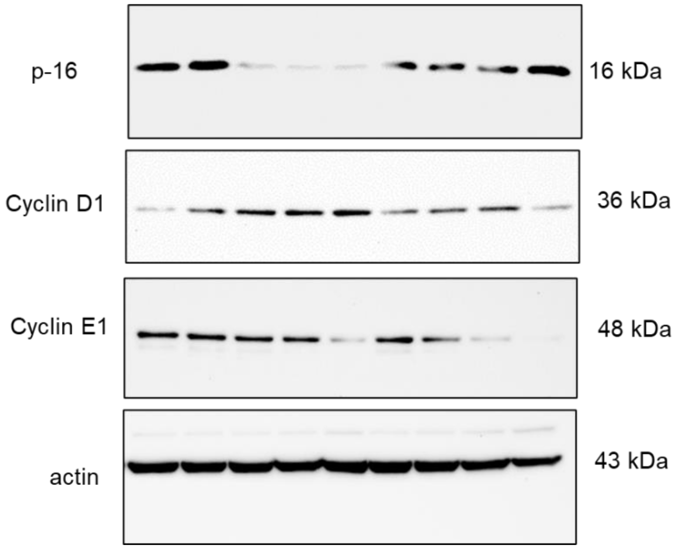

Figure 5B

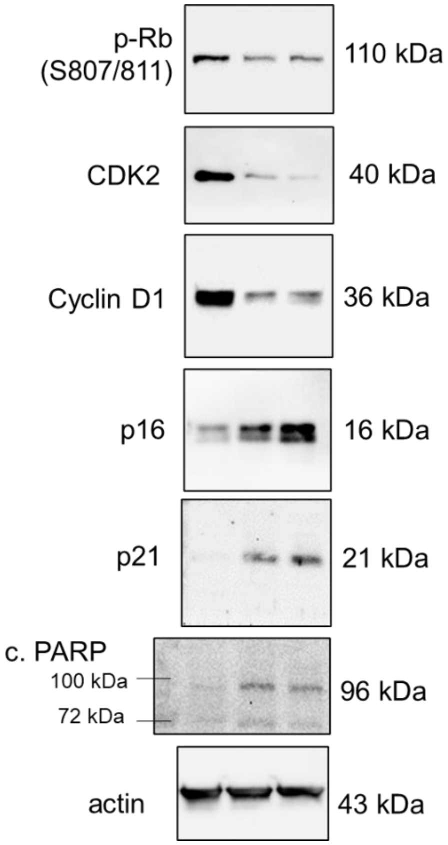

Figure 5C

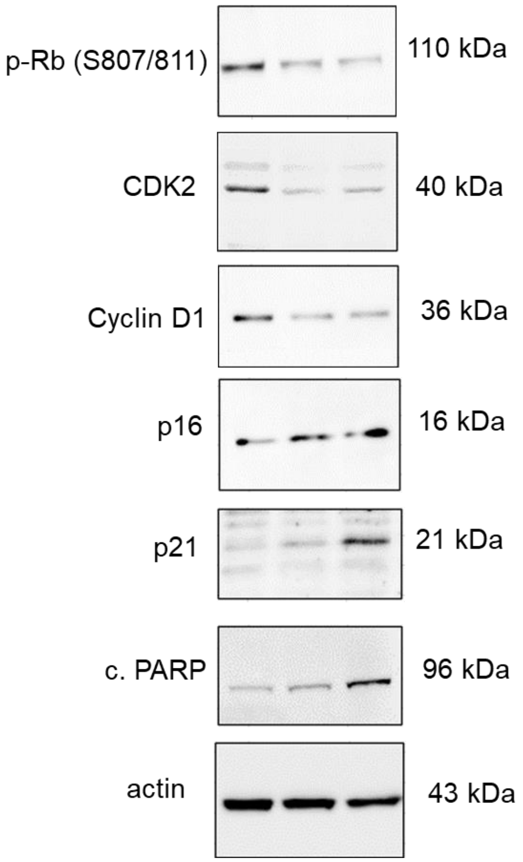

Figure 5H

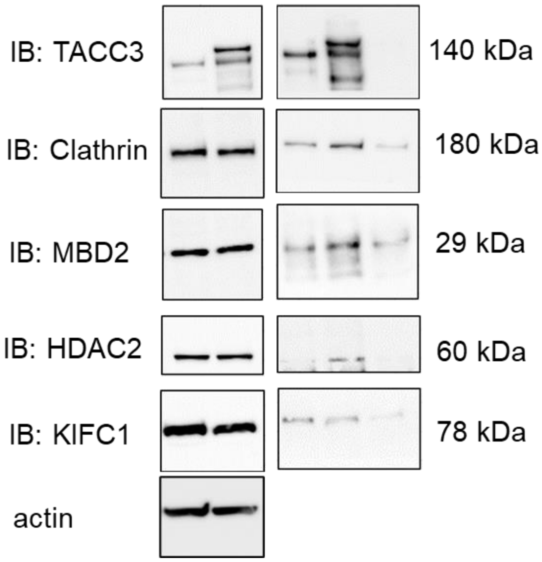

Figure 5I

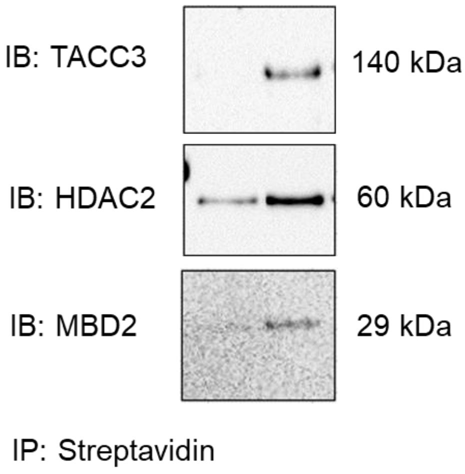

Figure 5J

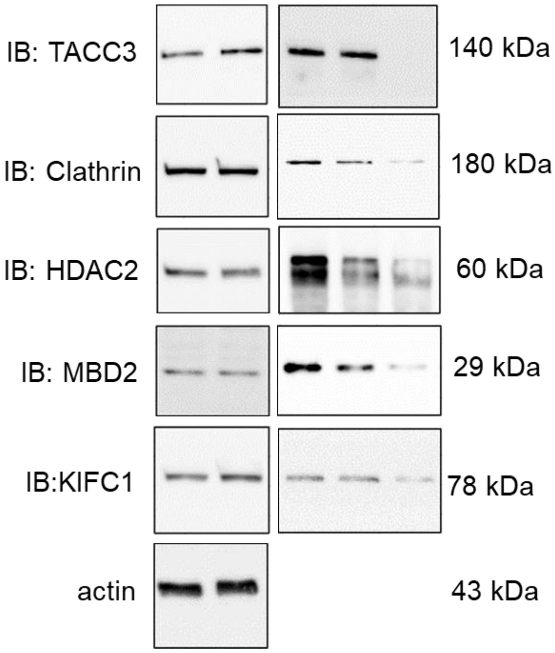

Figure 5K

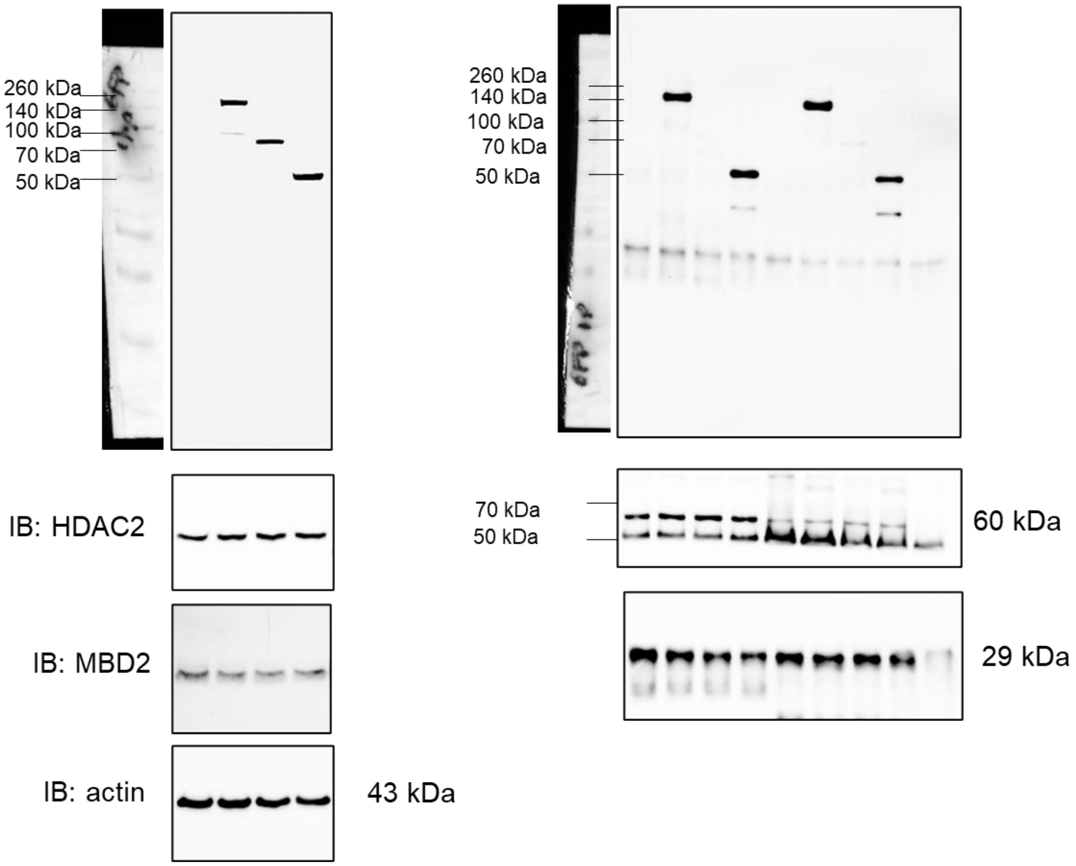

Figure 5P

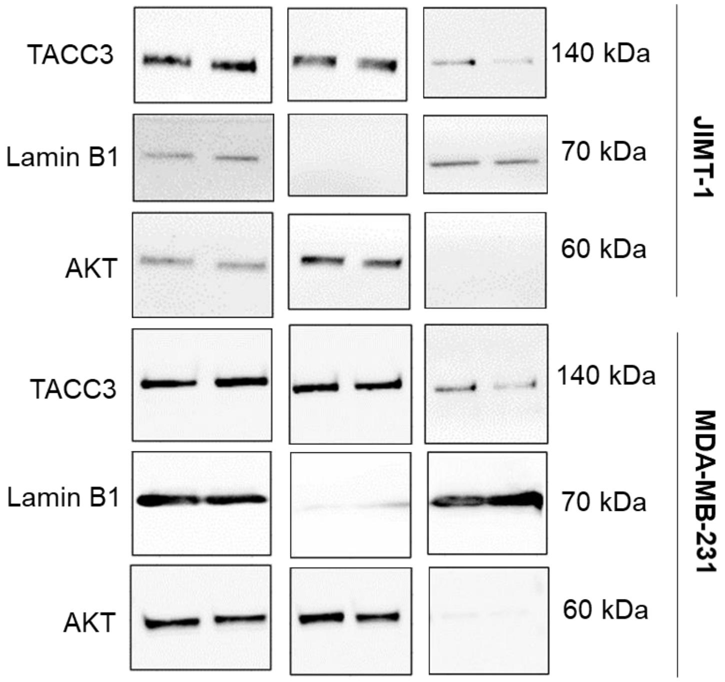

Figure 5Q

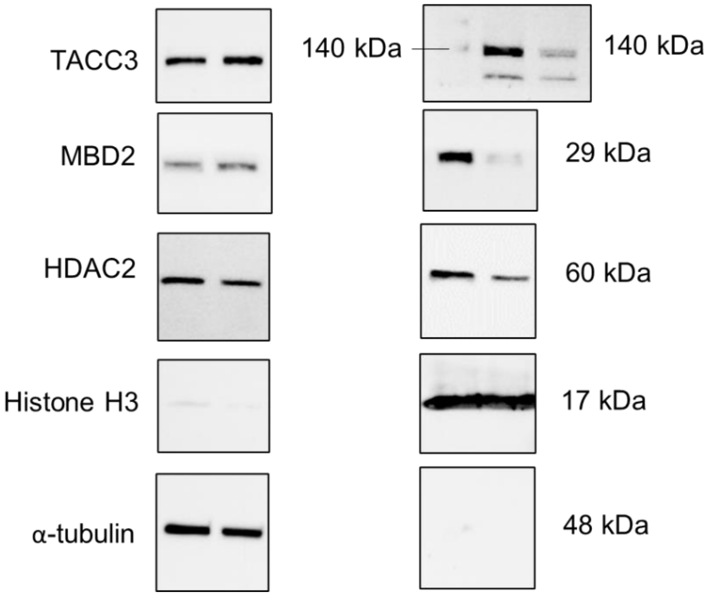

Figure 5R

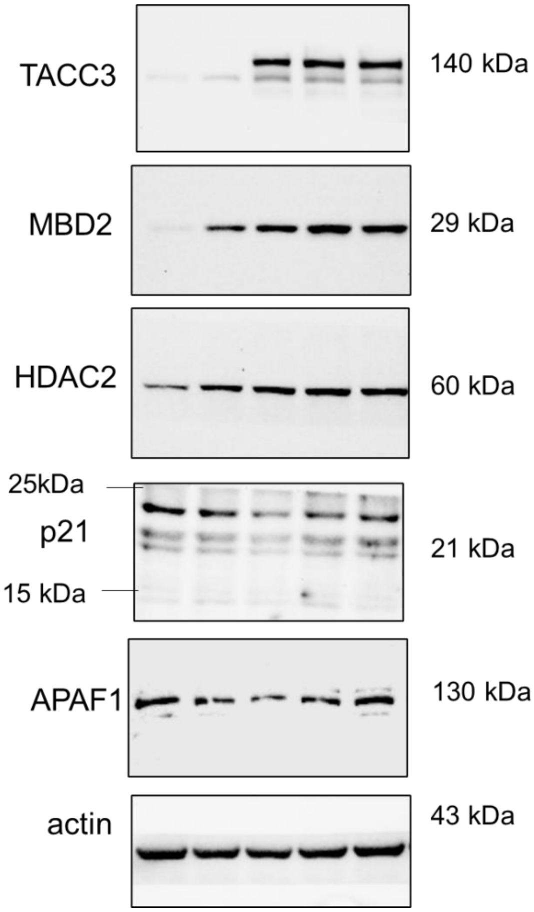

Figure 6A

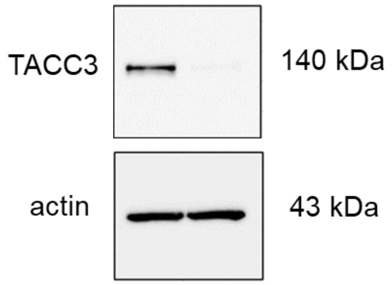

Figure 6M

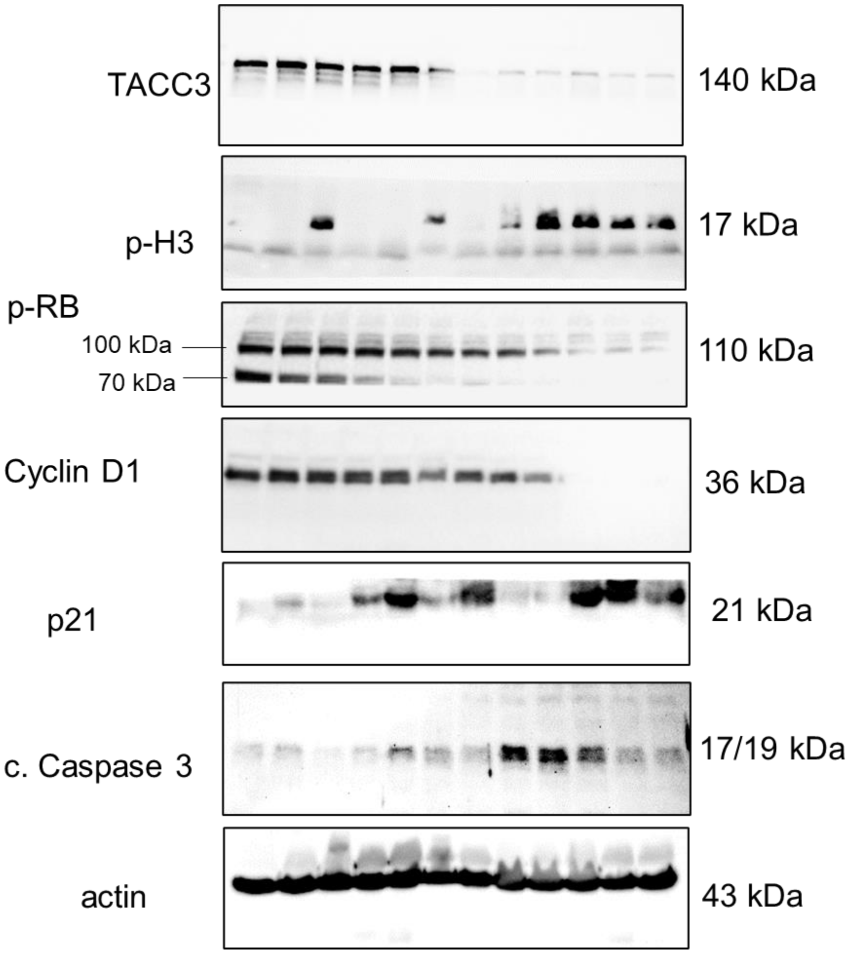

Figure S3A

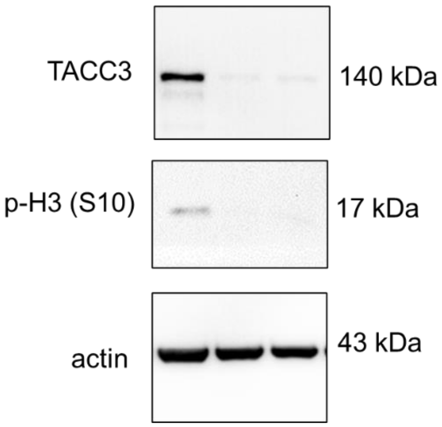

Figure S3F

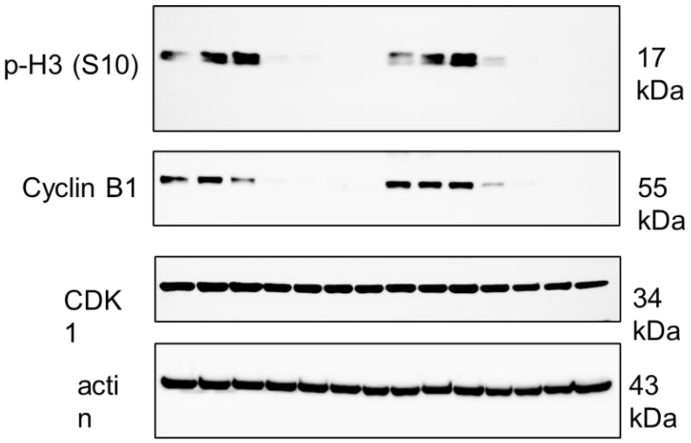

Figure S4C

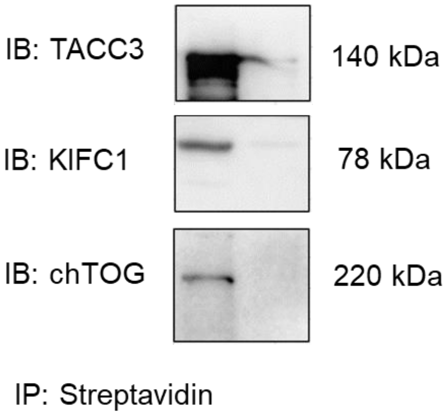

Figure S4D

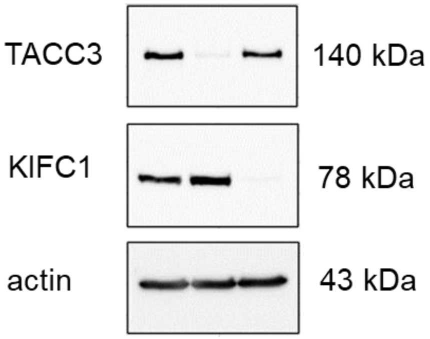

Figure S8A

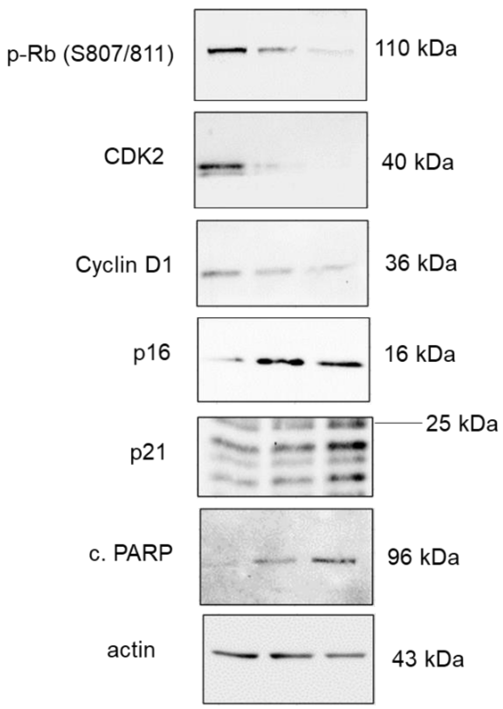

Figure S8B

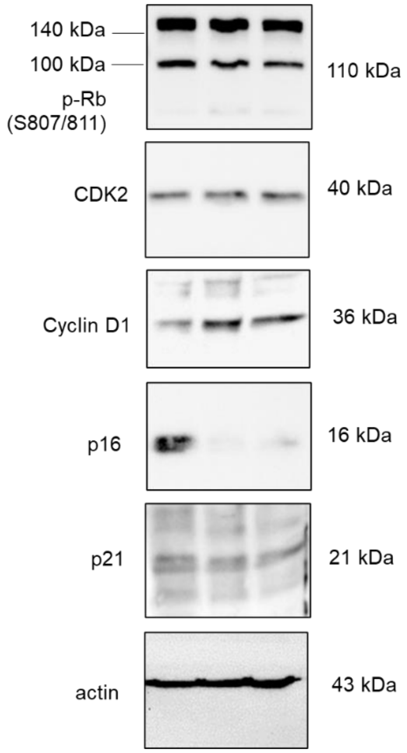

Figure S8F

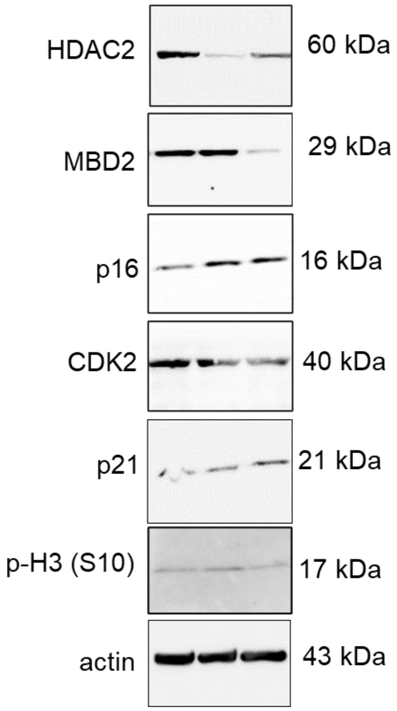

Figure S9A

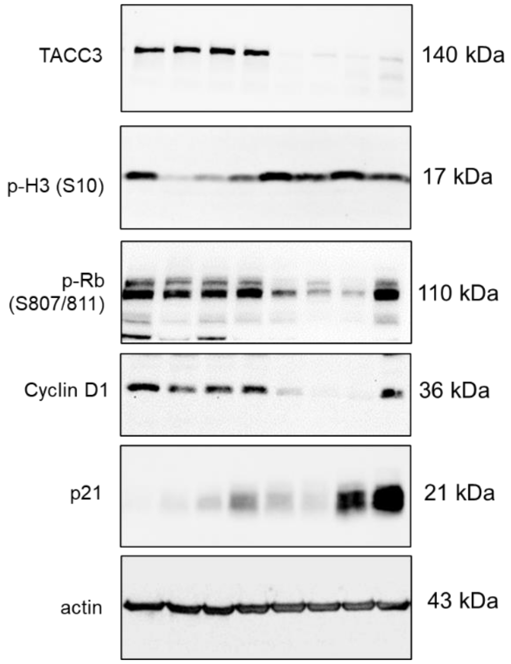

Figure S9B

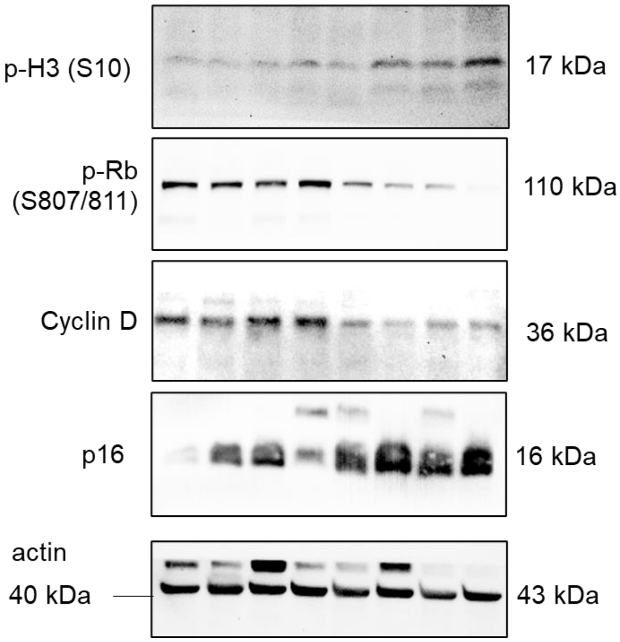

Figure S10H

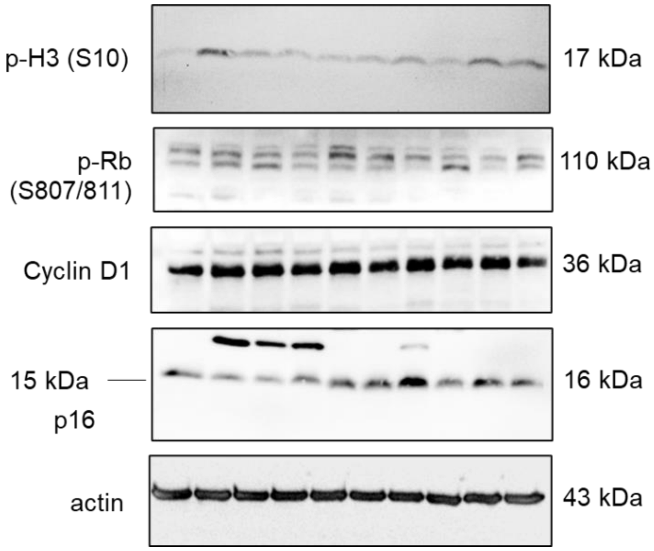

Supplement: Supplementary file 2 — Source Data for Western Blotting [file 41418_2023_1140_MOESM2_ESM.pdf]
